# Dual-mechanism vitamin C delivery by polyethylene glycol-23 glyceryl distearate-based niosomes via SVCT2 induction and enhanced transdermal penetration

**DOI:** 10.1080/10717544.2026.2681287

**Published:** 2026-05-30

**Authors:** Tatsuro Miyoshi, Brian C. Keller, Sui Nakagawa, Takashi Ashino, Asuka Kaizaki-Mitsumoto, Satoshi Numazawa

**Affiliations:** a Beverly Glen Laboratories, Inc., Newport Beach, CA, USA; b Department of Toxicology, Showa Medical University Graduate School of Pharmacy, Shinagawa, Tokyo, Japan

**Keywords:** GDS-23 niosomes, transdermal drug delivery, SVCT2, vitamin C, L-ascorbic acid, Nrf2 activation, antioxidant transporters, oxidative stress protection

## Abstract

Polyethylene glycol-23 glyceryl distearate (GDS-23)—a noisome-forming diacylglycerol–polyethylene glycol-based nonionic surfactant—functions as a nanocarrier for drug delivery systems (DDS) and an enhancer of endogenous antioxidant capacity through nuclear factor erythroid 2-related factor 2 (Nrf2) activation. Here, we evaluated the potential of GDS-23 as a multifunctional DDS carrier by investigating the combined contribution of (i) improved transdermal delivery mediated by niosomal DDS and (ii) increased cellular uptake associated with induction of antioxidant-related transporters, using protection against oxidative-stress-induced tissue damage as a functional readout. We determined whether GDS-23 modulates the expression of the antioxidant-related transporters xCT and SVCTs through Nrf2 activation. In normal human epidermal keratinocytes, GDS-23 treatment significantly upregulated the mRNA expression of *xCT* (*SLC7A11*; cystine/glutamate antiporter) and sodium-dependent vitamin C transporter 2 (*SVCT2*). The induction was diminished by an Nrf2 activation inhibitor (K67), indicating involvement of the Nrf2 pathway. Furthermore, evaluation using a three-dimensional reconstructed epidermis model with calcein sodium as a fluorescent tracer revealed that GDS-23-based niosomes facilitated skin permeation. When L-ascorbic acid or cysteine was encapsulated within these niosomes, hydroquinone-induced oxidative tissue damage was attenuated, with the L-ascorbic acid-loaded niosomes exhibiting the most prominent protective effect. Overall, this study shows that Nrf2 activation contributes to *SVCT2* induction and that GDS-23 is a multifunctional DDS nanocarrier that enhances skin permeation and may promote intracellular uptake by upregulating antioxidant-related transporters. These findings highlight the therapeutic potential of combining functional nanocarriers with entrapped bioactive molecules, offering a promising strategy for efficient treatment of skin disorders and antioxidant-based interventions.

## Graphical abstract

**Figure:**
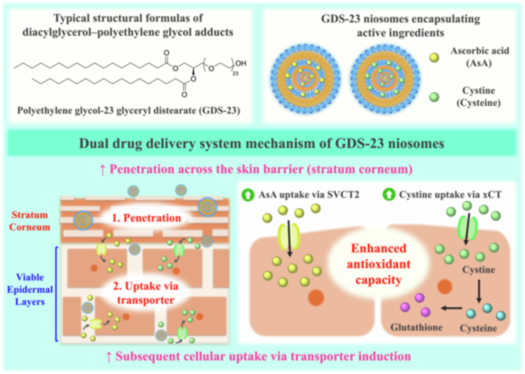

## Introduction

**Figure 1.**
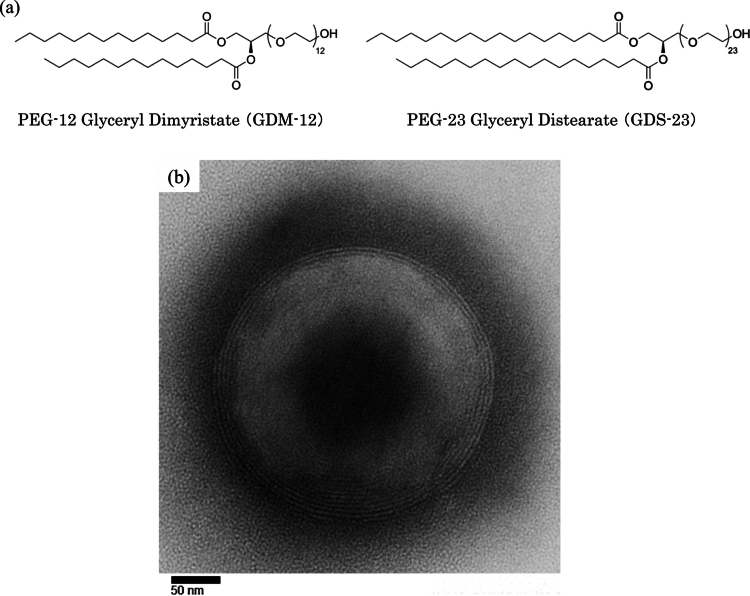
Structural features of diacylglycerol–polyethylene glycol adducts and their niosomes. (a) Typical structural formulas of diacylglycerol–polyethylene glycol adducts (GDM-12 and GDS-23). (b) Transmission electron microscopy (TEM) image of niosomes composed of the diacylglycerol–polyethylene glycol adduct.

Diacylglycerol–polyethylene glycol (PEG) adducts (Figure 1a) are nonionic surfactants capable of readily forming niosomes with multilamellar structures (Figure 1b) (Keller 2006). Niosomes are nanosized vesicles comprising nonionic surfactants and possess structural features similar to those of phospholipid-based liposomes. However, compared with liposomes, they offer several advantages, including easier and less costly preparation and higher stability (Seleci et al. 2016; Bartelds et al. 2018). Owing to their characteristic multilamellar organisation, with alternating hydrophilic and hydrophobic domains, niosomes can encapsulate hydrophilic and lipophilic compounds and have thus been investigated as versatile carriers for drug delivery systems (DDS) across a wide range of applications (Oku 2017; Moammeri et al. 2023; Lens 2025).

We previously investigated DDS using niosomes prepared from diacylglycerol–PEG adducts and demonstrated that they enhance transdermal drug permeation (Keller et al. 2010; Miyoshi et al. 2021). We reported that, beyond their role as drug carriers, these compounds activate nuclear factor erythroid 2-related factor 2 (Nrf2) through a non-canonical pathway involving p62 phosphorylation in human epidermal keratinocytes, thereby enhancing endogenous antioxidant defences (Miyoshi et al. 2023). In addition, they improve epidermal barrier function and moisture retention (Miyoshi et al. 2025). These diacylglycerol–PEG adducts (commercially known as QuSome®, a registered trademark of Beverly Glen Laboratories, Inc.; Figure 1a), including polyethylene glycol-12 glyceryl dimyristate (GDM-12) and polyethylene glycol-23 glyceryl distearate (GDS-23), self-assemble into nonionic surfactant-based niosomes. In the present study, we evaluated the potential of GDS-23, which demonstrated the most pronounced effects among these adducts, as a DDS material.

The skin, which forms the outermost surface of the human body, serves as a critical barrier against invading microorganisms, xenobiotics, oxidative stress, and transepidermal water loss (Bonté 2011; Ishitsuka et al. 2020; Rajkumar et al. 2023). Various technologies have been developed to overcome this barrier and improve transdermal drug delivery (Antonara et al. 2025; Sim et al. 2025). However, enhanced skin permeation alone does not ensure that therapeutic molecules reach their intracellular targets. Even after traversing the stratum corneum, additional barriers, such as the cell membrane, must be overcome before biological activity is achieved (Yameen et al. 2014; Liu et al. 2024). Recent studies have highlighted the efficacy of vesicle-based nanocarriers as promising platforms for drug delivery (Geng et al. 2025). In addition, extracellular vesicles are recognised as endogenous vesicles that mediate intercellular communication via transfer of bioactive molecules, such as RNA and proteins, and their potential applications in DDS have recently been explored (Lv et al. 2025). For example, tumour-derived exosomes enriched with Ras homologue family member A (RhoA) have been reported to promote transdermal delivery through transcytosis across epidermal cells, highlighting the potential of biologically derived vesicles for use as DDS platforms (Yang et al. 2025). Furthermore, ROS-responsive vesicle-based DDS have been reported to deliver antioxidant molecules as therapeutic strategies for oxidative stress–related diseases (Shen et al. 2025). In parallel, targeting DDS strategies have been developed in which nanocarriers are modified with PEG or ligands to improve biodistribution, local retention, and specificity toward target tissues or cells (Tachikawa et al. 2014; Sercombe et al. 2015; Gatto et al. 2024). These strategies highlight the importance of improving intracellular delivery; however, approaches that enhance cellular uptake by modulating the expression of transporters remain relatively underexplored.

In this context, we focused on the possibility that GDS-23, a niosome-forming diacylglycerol–PEG adduct, may enhance intracellular uptake through the modulation of specific membrane transporters. Small bioactive molecules, such as L-ascorbic acid (commonly known as vitamin C) and cystine, cannot efficiently permeate the cell membrane and require dedicated transporters—sodium-dependent L-ascorbic acid transporters (SVCT1 and SVCT2; collectively SVCTs) (Steiling et al. 2007; Savini et al. 2008) and the cystine/glutamate antiporter xCT (*SLC7A11*) (Lewerenz et al. 2013), respectively—for intracellular entry. The expression levels of these transporters strongly influence the intracellular availability and pharmacological activity of their substrates (Savini et al. 2008). Therefore, a DDS material that improves transdermal penetration and enhances transporter expression can offer a substantial therapeutic advantage.

We previously demonstrated that diacylglycerol–PEG adducts activate the Nrf2 pathway (Miyoshi et al. 2023), which, in turn, upregulates *xCT* expression as part of the antioxidant defence system, facilitating cystine uptake and promoting glutathione synthesis to protect cells from oxidative stress (Ye et al. 2014). In contrast, to the best of our knowledge, any such relationship between Nrf2 activation and *SVCT* regulation has not been reported. Nevertheless, L-ascorbic acid is an antioxidant molecule crucial for skin biology, which is present at higher concentrations in the epidermis than in the dermis, despite humans being unable to synthesise it endogenously (Steiling et al. 2007; Michalak et al. 2021).

SVCT-mediated transport is essential for maintaining cutaneous L-ascorbic acid levels. Beyond its antioxidant properties, L-ascorbic acid is a cofactor for collagen synthesis, regulates melanogenesis and keratinocyte differentiation, promotes wound healing, and contributes to multiple aspects of skin homoeostasis (Pullar et al. 2017). These findings highlight SVCT-mediated L-ascorbic acid transport as a promising therapeutic target for skin health and regenerative medicine. However, the regulatory mechanisms governing *SVCT* expression remain largely unclear.

In this study, we aimed to evaluate the potential of GDS-23 as a multifunctional DDS material that combines enhanced transdermal delivery with biological modulation of antioxidant-related transporters. We focused particularly on *SVCT2*, the more abundant isoform in the epidermis, which has a stronger affinity for L-ascorbic acid and plays a critical role in maintaining intracellular redox homoeostasis (Steiling et al. 2007; Savini et al. 2008; Ghahremani-Nasab et al. 2023). Finally, we determined whether the combination of GDS-23-mediated enhancement of skin penetration and transporter-mediated intracellular uptake contributes to protection against oxidative stress-induced tissue damage.

## Materials and methods

### Reagents

GDS-23 (polyethylene glycol-23 glyceryl distearate) and GDM-12 (polyethylene glycol-12 glyceryl dimyristate), proprietary materials developed by Beverly Glen Laboratories, Inc. (Newport Beach, CA, USA), were synthesised by NOF CORPORATION (Tokyo, Japan) and are not commercially available. L-ascorbic acid was obtained from DSM (Heerlen, The Netherlands; Code No. 0408050366). L-cysteine was obtained from FUJIFILM Wako Pure Chemical Corporation (Osaka, Japan; Cat. No. 039-20652). Hydroquinone was obtained from Junsei Chemical Co., Ltd. (Saitama, Japan; Cat. No. 59620). Calcein disodium salt was obtained from Fluka (Buchs, Switzerland; Cat. No. 21030-5G-F). Goat anti-rabbit IgG H&L (Alexa Fluor® 488) and goat anti-mouse IgG H&L (Alexa Fluor® 488) were purchased from Abcam (Cambridge, UK; Cat. Nos. ab150077 and ab150113, respectively). Cellstain® DAPI (4′,6-diamidino-2-phenylindole) solution was purchased from Dojindo Laboratories (Kumamoto, Japan; Cat. No. 340-07971). K67 (N,N′-(2-(2-oxopropyl)naphthalene-1,4-diyl)bis(4-ethoxybenzenesulfonamide); *N*-[2-acetonyl-4-(4-ethoxybenzenesulfonylamino)naphthalen-1-yl]-4-ethoxybenzenesulfonamide; 2-acetonyl-1,4-bis[(4-ethoxybenzenesulfonyl)amino]naphthalene) was purchased from Sigma-Aldrich (St. Louis, MO, USA; Cat. No. SML1922). Neutral Red Solution (0.33%) was also obtained from Sigma-Aldrich (Cat. No. N2889). 4% Paraformaldehyde phosphate buffer solution was obtained from FUJIFILM Wako Pure Chemical Corporation (Cat. No. 163-20145). Bovine serum albumin (BSA) was obtained from Sigma-Aldrich (Cat. No. A4503). Phosphate-buffered saline without calcium and magnesium (PBS(−)) was obtained from FUJIFILM Wako Pure Chemical Corporation (Cat. No. 166-23555). Citric acid and trisodium citrate was obtained from IWATA CHEMICAL CO., LTD. (Shizuoka, Japan; Reference Nos. CAM-G-01 and TSC-G-01, respectively).

PCR primer sets were purchased from Takara Bio (Shiga, Japan). The primer sets used in this study, along with their sequences, are listed in Table 1.

**Table 1.** Primers used in quantitative polymerase chain reaction.

Gene	Forward primer (5′–3′)	Reverse primer (5′–3′)
Glyceraldehyde-3-phosphate dehydrogenase (*GAPDH*)	GCACCGTCAAGGCTGAGAAC	TGGTGAAGACGCCAGTGGA
Cystine/glutamate antiporter (*xCT*)	CCCTATGCCAAACAGGTGAACA	GAAGACCCAATAAGTTTGCCGAAG
Sodium-dependent L-ascorbic acid transporters 2 (*SVCT2*)	TCCTGCAGTCAGAACCCGAGTA	GAATGTCTGTGAGGCTGCTGAA

All other reagents used in this study were of analytical grade.

### Cell culture

Normal human epidermal keratinocytes (NHEKs (NB); neonatal, primary human keratinocytes) were purchased from Kurabo (Tokyo, Japan; Cat. No. KK-4009, Lot No. 06228) and maintained in HuMedia-KG2 medium (Kurabo; Cat. No. KK-2150S) at 37 °C under 5% CO_2_. Cells were detached using 0.25% trypsin containing 0.01% EDTA (Kurabo; Cat. No. HK-3120) and reseeded onto culture plates for subsequent experiments. For studies on three-dimensional (3D) reconstructed epidermis, LabCyte EPI-MODEL 24 (Japan Tissue Engineering Co., Ltd., Aichi, Japan; Cat. No. 401124) and the SkinEthic™ RHE (Reconstructed Human Epidermis) model (EPISKIN, Lyon, France) were used. Each model was cultured in the manufacturer-supplied assay medium at 37 °C under 5% CO_2_.

### Quantitative real-time PCR (qPCR)

NHEKs were seeded at a density of 2 × 10^4^ cells/well in 96-well plates and cultured for 24 h in HuMedia-KG2. Based on our previous studies (Miyoshi et al. 2023, 2025), cells were then treated with HuMedia-KB2 (Kurabo; Cat. No. KK-2350S) containing 50 µM of GDS-23 for 18 or 24 h. For incubations exceeding 24 h, cells were post-cultured in HuMedia-KB2 alone for an additional 6 h (total 30 h), to account for the temporal dynamics of Nrf2 activation. Time points are indicated with the initiation of GDS-23 treatment, defined as 0 h. Control cells were cultured in HuMedia-KB2 for the indicated time periods.

Total RNA was extracted using the RNeasy Mini Kit (Qiagen, Hilden, Germany; Cat. No. 74106), and cDNA was synthesised using ReverTra Ace qPCR RT Master Mix (Toyobo, Osaka, Japan; Cat. No. FSQ-201) with a PCR Thermal Cycler Dice system (Takara Bio).

qPCR was performed using SYBR Green Master Mix (Thermo Fisher Scientific, Waltham, MA, USA; Cat. No. A25742) on a StepOne Real-Time PCR System (Applied Biosystems, Thermo Fisher Scientific, Waltham, MA, USA). Relative mRNA levels were calculated using the ΔΔCt method, with *GAPDH* serving as the internal control.

### qPCR with Nrf2 inhibition

NHEKs were seeded at 2 × 10^4^ cells/well in 96-well plates and cultured for 8 h in HuMedia-KG2. Cells were treated with HuMedia-KB2 containing 25 μM K67, a concentration determined based on our previous studies (Miyoshi et al. 2025), for 16 h, followed by treatment with HuMedia-KB2 containing 50 μM GDS-23 for 24 h. Control cells were cultured in HuMedia-KB2 for 24 h. RNA extraction, cDNA synthesis, and qPCR analysis were performed as described above, using *GAPDH* as the internal control.

### Hydroquinone-induced cytotoxicity assay

NHEKs were seeded at 2 × 10^4^ cells/well in 96-well plates, cultured in HuMedia-KG2 for 24 h, and treated sequentially in HuMedia-KB2 as follows:

(1) GDS-23-containing medium (or HuMedia-KB2 alone) for 24 h;
(2) L-ascorbic acid- or cysteine-containing medium (or HuMedia-KB2 alone) for 24 h;
(3) 0.2% hydroquinone-containing medium (or HuMedia-KB2 alone) for 24 h.

These three-step treatments were applied in the following combinations:

- GDS-23 → L-ascorbic acid or cysteine → hydroquinone
- GDS-23 → untreated → hydroquinone
- Untreated → L-ascorbic acid or cysteine → hydroquinone
- Untreated → untreated → hydroquinone
- Untreated → untreated → untreated (control)

Following treatment, cells were incubated with HuMedia-KB2 containing 33 µg/mL Neutral Red for 2 h. Internalised dye was extracted with 0.1 N HCl in 30% ethanol, and absorbance was measured at 550 nm, with background subtraction at 650 nm. Cell viability was calculated based on absorbance values.

### Characterisation of GDS-23 niosomes

#### Transmission electron microscopy (TEM)

Niosome samples prepared from GDS-23 were diluted appropriately and applied to carbon-coated copper grids for 30 s. Excess liquid was removed with filter paper, and grids were partially dried, stained with 2% phosphotungstic acid for 10 s at 80 °C, blotted again, and air-dried. Images were acquired using a JEM-1200EX transmission electron microscope (JEOL Ltd., Tokyo, Japan) at an acceleration voltage of 80 kV.

#### Dynamic light scattering (DLS)

Particle size was analysed using a Litesizer™ 500 instrument (Anton Paar GmbH, Graz, Austria) equipped with a 40 mW diode laser (*λ* = 658 nm). Samples were diluted 10-fold with purified water and measured at 25 °C. Hydrodynamic diameters (intensity-weighted averages) were obtained using cumulant analysis (ISO 22412), and particle size distributions were assessed using non-negative least squares analysis.

### Evaluation of transdermal penetration using 3D epidermis

Transdermal permeation was evaluated using the SkinEthic™ RHE model, a standardised reconstructed epidermis model compliant with the OECD Test Guideline TG 428 (Schäfer-Korting et al. 2006). After overnight equilibration at 37 °C in assay medium, 150 µL of either a 2.0% GDS-23 solution containing 0.1% calcein sodium or a 0.1% calcein sodium solution was applied to the apical surface and incubated for 6 h. Calcein sodium was then extracted from the tissues, and fluorescence was measured (excitation: 494 nm; emission: 520 nm). Permeated calcein sodium was quantified using a standard calibration curve.

For fluorescence imaging, tissues were embedded in Tissue-Tek® O.C.T. Compound (Sakura Finetek Japan Co., Ltd., Tokyo, Japan), snap-frozen in liquid nitrogen, and sectioned at 5 µm. The sections were rinsed with purified water to remove residual O.C.T. compound and examined using a fluorescence microscope (BZ-X810, Keyence Corp., Osaka, Japan). For nuclear staining, the sections were incubated with 200-fold diluted DAPI in 3% BSA/PBS (−) for 10 min in the dark, rinsed with PBS(−), and subsequently imaged.

### Immunofluorescence staining using 3D epidermis

The LabCyte EPI-MODEL 24, a reconstructed human epidermis model compliant with OECD TG 431 and TG 439 and widely used for immunohistology (Katoh et al. 2009a, 2009b; OECD 2025a, 2025b), was used. After overnight equilibration at 37 °C, 50 µL of either 2% GDS-23 solution (pH 5.5; adjusted with 0.02% citric acid and 0.08% trisodium citrate) or purified water (pH 5.6) was applied to the apical surface and incubated for 6 h. The surface was rinsed, and the tissues were incubated for an additional 24 h without further treatment. Samples were embedded in O.C.T. compound, frozen, and sectioned at 5 µm.

The sections were fixed with 4% paraformaldehyde for 5 min, rinsed with PBS(-), incubated with NH_4_Cl for 10 min, and blocked for 1 h in 3% BSA/PBS containing 10% normal goat serum. After removal of the blocking solution, the sections were incubated with primary antibodies diluted in 3% BSA/PBS for 2 h at room temperature (25 ± 3 °C). After washing with PBS(-), the sections were incubated with Alexa Fluor® 488-conjugated secondary antibodies for 1 h in the dark. The nuclei were stained with DAPI (200-fold dilution in 3% BSA/PBS) for 10 min. After final washing, the samples were mounted and imaged via fluorescence microscopy.

The following primary antibodies were used: *xCT* (*SLC7A11*) rabbit polyclonal antibody (Proteintech, Rosemont, IL, USA; Cat. No. 26864-1-AP) and *SVCT2* rabbit polyclonal antibody (Proteintech, Cat. No. 27019-1-AP), both diluted 1:100. The following secondary antibody was used: Goat anti-rabbit IgG H&L (Alexa Fluor® 488) (Abcam, Cat. No. ab150077), diluted 1:500.

### Hematoxylin and eosin (H&E) staining

Frozen tissue sections were mounted onto glass slides, fixed with 4% paraformaldehyde for 5 min, and rinsed with purified water. The sections were stained using the Hematoxylin and Eosin Stain Kit (ScyTek Laboratories, Logan, UT, USA; Cat. No. HAE-1).

Clearing was performed by sequential immersion in a 1:1 ethanol–xylene solution for 5 min, followed by three changes of xylene (5 min each). Slides were mounted using a permanent mounting medium, dried, and examined under a microscope.

### Assessment of hydroquinone-induced tissue damage using a 3D reconstructed epidermis model

Hydroquinone-induced tissue damage was evaluated using the LabCyte EPI-MODEL 24, a reconstructed human epidermis model that exhibits high structural and functional similarity to native human epidermis and is compliant with the OECD Test Guideline 439 (OECD TG 439; OECD 2025b). The treatment schedule and composition of each test sample are summarised in Table 2.

**Table 2.** Experimental design for hydroquinone-induced tissue damage evaluation.

Sample no.	Sample	For 6 h each on days 1–3 and 6–8	Hydroquinone (HQ) Treatment (2 h)	pH
1	Control	Purified water	Purified water	5.6
2	Hydroquinone (HQ)	Purified water	0.2% HQ aqueous solution	5.6
3	HQ + GDS-23	2% GDS-23 niosome dispersion	0.2% HQ aqueous solution	5.5
4	HQ + L-Cysteine (Cys)	0.2% Cys aqueous solution	0.2% HQ aqueous solution	5.6
5	HQ + L-Ascorbic Acid (AsA)	0.2% AsA aqueous solution	0.2% HQ aqueous solution	5.5
6	HQ + AsA + Cys	0.2% AsA + 0.2% Cys aqueous solution	0.2% HQ aqueous solution	5.5
7	HQ + GDS-23 + Cys	0.2% Cys encapsulated in 2% GDS-23 niosome dispersion	0.2% HQ aqueous solution	5.6
8	HQ + GDS-23 + AsA	0.2% AsA encapsulated in 2% GDS-23 niosome dispersion	0.2% HQ aqueous solution	5.4
9	HQ + GDS-23 + AsA + Cys	0.2% AsA + 0.2% Cys encapsulated in 2% GDS-23 niosome dispersion	0.2% HQ aqueous solution	5.5

For each treatment cycle, 50 μL of the test solution was applied to the stratum corneum side of the epidermal surface, and the sample was incubated for 6 h. The tissues were then rinsed with purified water and incubated for an additional 18 h without treatment. This 24 h cycle (6 h treatment + 18 h recovery) was repeated for 3 consecutive days. The tissues were then cultured for 2 days without treatment, followed by one additional treatment cycle consisting of a 6 h exposure to 50 μL of the test solution and an 18 h recovery period, repeated for 2 days.

On day 8, tissues were treated with the respective test solutions for 6 h, rinsed, and subsequently exposed to 0.2% hydroquinone aqueous solution for 2 h. After hydroquinone treatment, tissues were rinsed again and cultured for an additional 2 days without treatment. On day 10, the tissues were embedded, frozen, and sectioned for H&E staining. During the entire culture period, the medium on the receiver side was replaced every 2 days.

The pH of all test solutions was adjusted to 5.5 ± 0.1 with citric acid and trisodium citrate. The measured pH values are listed in Table 2.

Quantitative analysis of tissue damage was performed using the BZ-X Analyser software (Keyence Corp., Osaka, Japan). Vacuole (void) areas within the viable epidermal layers were quantified, and the percentage of void area relative to the total viable epidermis area was calculated as the tissue-damage index.

### Statistical analysis

Statistical analyses were performed using the JMP Pro 16 software (SAS Institute Japan, Tokyo, Japan). Data are expressed as mean ± S.D. A Student's *t*-test was used to compare the control group with each experimental group. One-way analysis of variance with post-hoc Tukey's test was used for multiple comparisons among three or more groups. *p < 0.05, **p < 0.01, and ***p < 0.001 represent statistical significance.

## Ethical approval statement

This study did not involve humans or animals. All experiments were conducted using commercially available human cells and reconstructed human epidermis models. Therefore, no institutional review board approval was required.

## Results

### Induction of *xCT* and *SVCT2* gene expression by GDS-23 and involvement of Nrf2 activation

**Figure 2.**
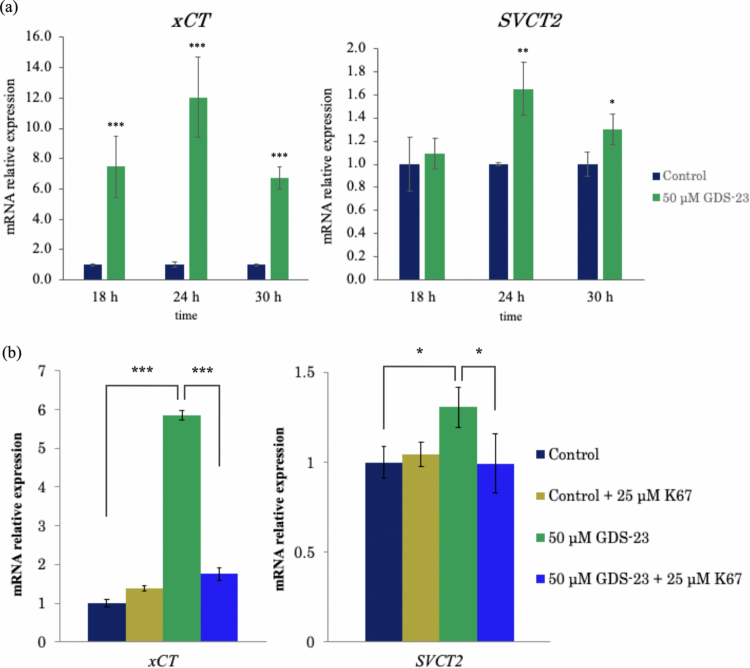
Effects of GDS-23 on *xCT* and *SVCT2* mRNA expression and involvement of Nrf2 activation. (a) NHEKs were treated with 50 µM GDS-23 for 18 or 24 h or with 50 µM GDS-23 for 24 h followed by incubation in culture medium alone for an additional 6 h (total 30 h). Control cells were incubated with culture medium alone. mRNA levels of *xCT* and *SVCT2* were quantified using quantitative PCR (qPCR). Data are presented as mean ± standard deviation (*n* = 4). Statistical analysis was performed using Student’s *t*-test; *p < 0.05, **p < 0.01, and ***p < 0.001 vs. control). (b) NHEKs were pretreated with either 25 µM K67 or culture medium for 16 h, followed by treatment with 50 µM GDS-23 for 24 h. Control cells were incubated with culture medium alone. mRNA levels were quantified as described in (a). Data are presented as mean ± standard deviation (*n* = 4). Statistical analysis was performed using Tukey’s test; *p < 0.05, **p < 0.01, and ***p < 0.001.

We first examined the effects of GDS-23 on the mRNA expression of *xCT* and *SVCT2* in NHEKs. GDS-23 significantly upregulated the mRNA levels of both *xCT* and *SVCT2* (Figure 2a) and increased the mRNA expression of *SVCT1*. Treatment with GDM-12, a structurally related diacylglycerol–PEG adduct, similarly upregulated the mRNA expression of *xCT* and *SVCT2* (Figures S1 and S2).

Next, we determined whether Nrf2 activation contributes to the GDS-23-induced upregulation of these transporters. Consistent with our previous report, GDS-23 activates Nrf2 via a non-canonical pathway mediated by phosphorylation of p62 (Miyoshi et al. 2023).

Phosphorylated p62 enhances its interaction with Keap1, thereby promoting nuclear translocation of Nrf2 and transcriptional activation of downstream target genes (Ichimura et al. 2013). In this study, we employed K67, a compound known to inhibit the interaction between phosphorylated p62 and Keap1 (Saito et al. 2016). NHEKs were pretreated with K67 for 16 h, followed by GDS-23 exposure for 24 h. Under these conditions, the induction of *xCT* and *SVCT2* mRNA expression by GDS-23 was significantly attenuated (Figure 2b). These findings support the involvement of the non-canonical Nrf2 activation pathway in the GDS-23-mediated upregulation of *xCT* and *SVCT2*.

### Upregulation of *xCT* and *SVCT2* by GDS-23 is associated with intracellular uptake of cysteine and L-ascorbic acid

To determine whether GDS-23-induced upregulation of *xCT* and *SVCT2* is associated with enhanced intracellular uptake of cysteine and L-ascorbic acid, we evaluated their protective effects against hydroquinone-induced cytotoxicity. Although hydroquinone is an effective depigmenting agent, it is rapidly oxidised to highly toxic metabolites, such as *p*-benzoquinone and hydroxybenzoquinone, resulting in local cytotoxicity (Nordlund et al. 2006; Draelos 2007). In our previous study (Miyoshi et al. 2023) and in preliminary experiments (Figure S3), hydroquinone-induced cytotoxicity was mitigated either by preventing hydroquinone oxidation through the co-presence of antioxidants in the medium or by enhancing intracellular antioxidant capacity.

In the present study, NHEKs were pretreated with GDS-23 to increase transporter expression and then exposed to low concentrations of cysteine or L-ascorbic acid, followed by hydroquinone treatment, after which cell viability was assessed.

As cystine is poorly soluble in aqueous media, its reduced form, cysteine, was used. Cysteine is rapidly oxidised extracellularly to cystine, which is taken up via xCT and subsequently reduced intracellularly, where it contributes to glutathione synthesis and antioxidant defence (Conrad and Sato 2012; Lewerenz et al. 2013; Ye et al. 2014). Thus, cysteine was added with the expectation that it would be converted to cystine in the extracellular environment.

**Figure 3.**
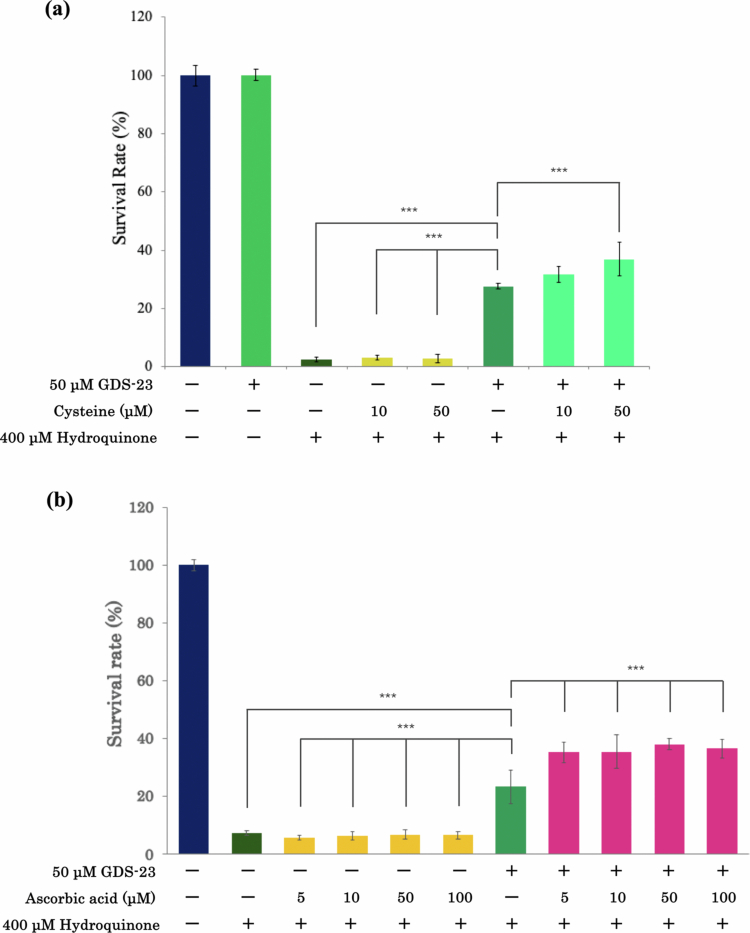
Protective effects of GDS-23-induced *xCT* and *SVCT2* upregulation on hydroquinone-induced cytotoxicity via enhanced uptake of cysteine or L-ascorbic acid. (a) NHEKs were pretreated with 50 µM GDS-23 for 24 h, followed by pretreatment with cysteine for an additional 24 h, and subsequently exposed to 400 µM hydroquinone for 24 h. For comparison, cells treated with hydroquinone alone (400 µM, 24 h) and cells pretreated with cysteine for 24 h prior to hydroquinone exposure were included. (b) NHEKs were pretreated with 50 µM GDS-23 for 24 h, followed by pretreatment with L-ascorbic acid for 24 h and 400 µM hydroquinone for 24 h. Cells treated with hydroquinone alone and those pretreated with L-ascorbic acid prior to hydroquinone exposure were used as comparison groups. For both (a) and (b), data are presented as mean ± standard deviation (*n* = 6). Statistical analysis was performed using Tukey’s test; *p < 0.05, **p < 0.01, and ***p < 0.001.

Neither cysteine nor L-ascorbic acid alone protected against hydroquinone-induced cytotoxicity (Figure 3a,b). Consistent with our previous findings (Miyoshi et al. 2023), pretreatment with 50 µM GDS-23 significantly improved cell viability (Figure 3a,b). Importantly, sequential treatment with GDS-23 followed by either cysteine or L-ascorbic acid resulted in greater cytoprotection than treatment with GDS-23 alone, with the L-ascorbic acid group exhibiting the most pronounced improvement in cell viability (Figure 3a,b). Notably, L-ascorbic acid exerted its protective effect at lower concentrations than did cysteine, despite *SVCT2* having a lower induction level than *xCT*, indicating a higher transport efficiency of *SVCT2*. These results suggest that GDS-23-induced upregulation of *xCT* and *SVCT2* may contribute to enhanced intracellular uptake of cysteine (cystine) and L-ascorbic acid, thereby increasing antioxidant activity and further attenuating hydroquinone-induced cytotoxicity.

### Structural characterisation of GDS-23 niosomes and evaluation of transdermal delivery using a 3D epidermis model

Next, we characterised the structural properties of niosomes formed by GDS-23. TEM revealed that GDS-23 niosomes exhibited a multilamellar vesicular structure (Figure 4a). DLS analysis showed that particle sizes were predominantly within the 100–200 nm range (Figure 4b). These findings confirmed that the GDS-23-derived niosomes used in this study possessed a multilamellar architecture and had particle diameters of 100–200 nm.

Figure 4.Structural characterisation of 2% GDS-23 niosomes and percutaneous absorption assessment using a three-dimensional cultured epidermal model (SkinEthic™ RHE). (a) Transmission electron microscopy (TEM) images. (b) Particle size distribution measured via dynamic light scattering (DLS). (c) Percutaneous absorption assay (quantitative analysis): From the stratum corneum side of the SkinEthic™ RHE tissues, 150 µL of either 2.0% GDS-23 aqueous solution containing 0.1% calcein sodium or 0.1% calcein sodium aqueous solution alone was applied and incubated for 6 h. After treatment, calcein sodium was extracted from the tissues, and fluorescence intensity (excitation: 494 nm, emission: 520 nm) was measured. The amount of permeated calcein sodium was determined from a standard calibration curve, and the results were expressed as a ratio relative to the control. Data are shown as mean ± standard deviation (*n* = 4). Statistical significance was determined using Student’s *t*-test (*p < 0.05, **p < 0.01, ***p < 0.001 vs. control). (d) Percutaneous absorption assay (fluorescence imaging): Tissues treated under the same conditions as in (c) were frozen, and cryosections were prepared for fluorescence microscopy. ‘Fluorescence-1’ shows the calcein fluorescence signal alone, and ‘Fluorescence-2’ shows merged images of calcein fluorescence and DAPI nuclear staining.Figure showing characterization and skin-penetration performance of GDS-23 nanoparticles. The top panels present nanoparticle morphology and size analysis: a transmission electron micrograph reveals spherical, multilamellar vesicle-like particles with concentric ring structures and diameters in the nanometer range (scale bar 200 nm), while dynamic light scattering data show a narrow particle-size distribution centered around 155–164 nm with an average diameter of 160.78 nm across three measurements. The lower panels evaluate penetration through a reconstructed human epidermis model. A bar graph demonstrates significantly enhanced calcein sodium salt penetration in the presence of 2% GDS-23, showing approximately threefold greater relative penetration than the control. Representative fluorescence microscopy images of skin cross-sections corroborate these findings, displaying stronger and deeper green fluorescence throughout the epidermal layers in the 2% GDS-23-treated samples compared with the weaker signal in controls; merged images with DAPI-stained nuclei (blue) further highlight increased penetration and distribution of the fluorescent marker (scale bars 50 μm).

**Figure:**
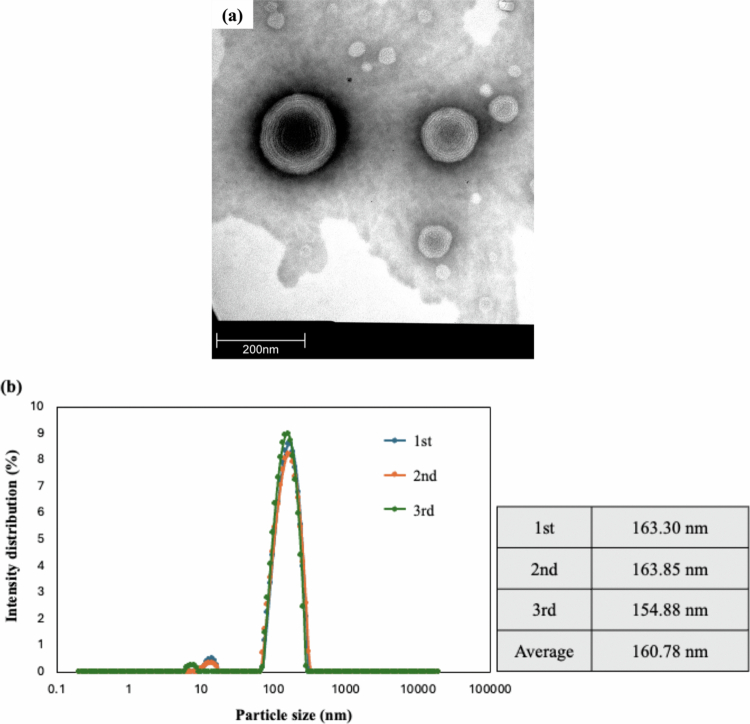

**Figure:**
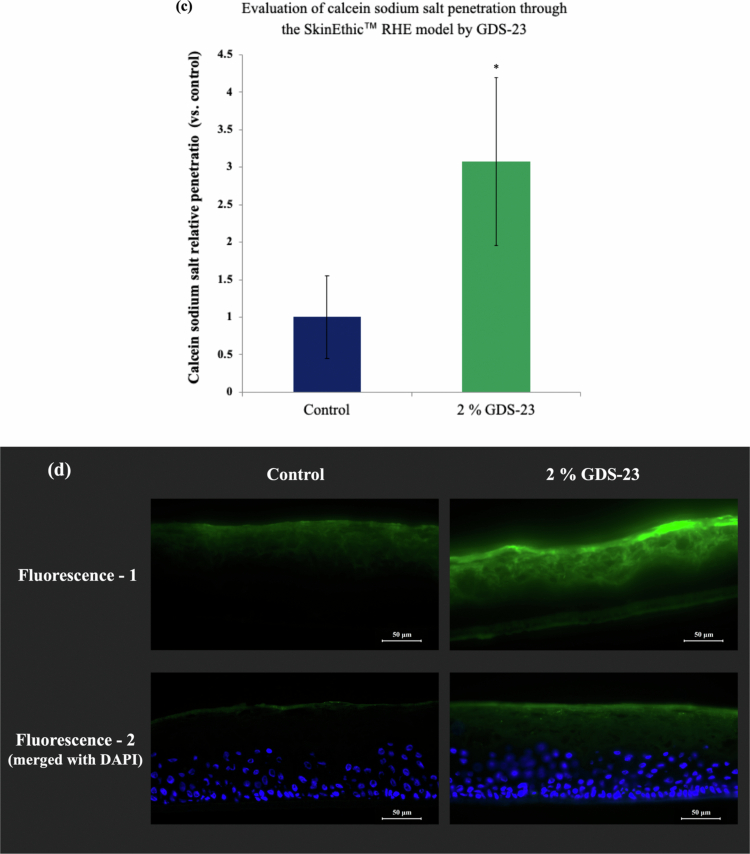

To evaluate transdermal delivery capability, GDS-23 niosomes encapsulating calcein sodium were applied to the stratum corneum side of the SkinEthic™ RHE 3D epidermis model. Based on our previous work (Miyoshi et al. 2025), the incubation period was set to 6 h. Compared with the calcein sodium aqueous solution control, the GDS-23 niosome treatment resulted in significantly greater permeation.

Penetration of calcein sodium in the GDS-23 niosome group was significantly higher than in the solution control, with a 3.07 ± 1.12-fold increase (*n* = 4, *p* = 0.046) (Figure 4c). Fluorescence imaging further revealed that fluorescence in the control group was confined mainly to the upper stratum corneum, whereas the GDS-23 niosome group exhibited fluorescence signals extending into deeper stratum corneum layers and approaching the viable epidermis (Figure 4d). These observations indicate that multilamellar niosomes formed by GDS-23 effectively enhance skin permeation and serve as potent carriers for transdermal drug delivery.

As supplementary data, niosomes formed from GDM-12, another diacylglycerol–PEG adduct structurally related to GDS-23, also exhibited multilamellar structures and particle sizes within the 100–200 nm range. Consistent with GDS-23, GDM-12 niosomes exhibited significantly enhanced permeability in the SkinEthic™ RHE model compared with the aqueous solution control (Figure S4 a–c).

### GDS-23 niosomes enhance *xCT* and *SVCT2* expression in a 3D reconstructed epidermis model

**Figure 5.**
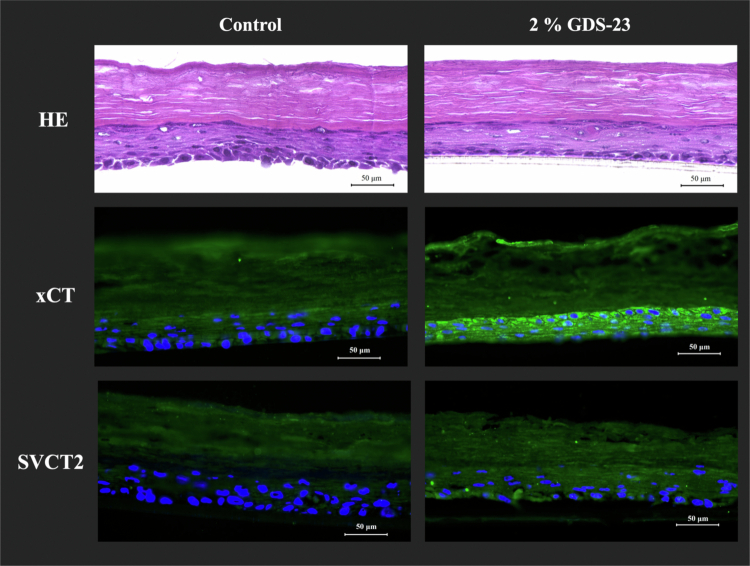
Hematoxylin and eosin (H&E) and immunofluorescence staining of *xCT* or *SVCT2* in 3D-cultured epidermal tissues (LabCyte EPI-MODEL 24) treated with 2% GDS-23. The three-dimensional cultured epidermal model (LabCyte EPI-MODEL 24) was treated from the stratum corneum side with 2% GDS-23 aqueous solution for 6 h. After removal of GDS-23 from the surface, tissues were post-incubated for 24 h. Frozen sections were prepared and stained with H&E or immunofluorescence for xCT or SVCT2.

We examined whether the GDS-23-induced upregulation of *xCT* and *SVCT2* observed in NHEKs also occurred in a 3D reconstructed human epidermis model (LabCyte EPI-MODEL 24). Based on our previous report (Miyoshi et al. 2025), tissues were treated with 2% GDS-23 niosomes from the stratum corneum side for 6 h, followed by 24 h of post-incubation after niosome removal. Immunofluorescence imaging revealed increased fluorescence signals corresponding to xCT and SVCT2 in GDS-23-treated tissues compared with those in control tissues (Figure 5). These findings indicate that GDS-23 niosomes are associated with increased protein expression of *xCT* and *SVCT2* in the 3D epidermis model.

### Evaluation of GDS-23-induced *xCT* and *SVCT2* upregulation and the resulting enhancement of cysteine and ascorbic acid uptake in a 3D epidermis model

Consistent with the NHEK results, GDS-23 treatment increased the expression of *xCT* and *SVCT2* in the 3D epidermal model (Figure 5). We, therefore, examined whether this upregulation enhanced the intracellular uptake of cysteine and L-ascorbic acid and promoted protection against hydroquinone-induced tissue damage, using the same indicator applied to monolayer cultures. To allow sufficient time for transporter induction and metabolite uptake, a 10-day culture schedule was employed (Table 2, Figure 6a). Each treatment cycle consisted of a 6 h topical application of test solutions to the stratum corneum surface, followed by rinsing and an 18-hour recovery period. This cycle was repeated for 3 days, followed by 2 days of untreated culture. A second 3-day treatment cycle was then performed, and on day 8 (the final day of treatment), tissues were exposed to 0.2% hydroquinone for 2 h immediately after the 6 h treatment. After removal of hydroquinone, tissues were rinsed and incubated without treatment for an additional 2 days. Frozen tissue sections were prepared and stained with H&E to assess tissue morphology.

Figure 6.Evaluation of hydroquinone-induced tissue damage in 3D-cultured epidermal tissues (LabCyte EPI-MODEL 24) treated with 2% GDS-23. (a) Experimental protocol. (b) Hematoxylin and eosin (H&E)-stained images of three-dimensional cultured epidermal tissues (LabCyte EPI-MODEL 24) treated according to the experimental design shown in (a) and Table 2. Images are focused on the viable epidermal layers, with void areas representing tissue damage highlighted in yellow. (c) Quantitative analysis of void areas detected in (b). The void area ratio was calculated as the percentage of the void area relative to the total area of the viable epidermal layer. Data are presented as mean ± standard deviation (*n* = 4). Statistical significance was determined using Tukey’s *t*-test (*p < 0.05, **p < 0.01, ***p < 0.001).Composite figure showing a 3D epidermis experimental workflow, representative H&E-stained tissue sections, and quantitative analysis of hydroquinone (HQ)-induced damage. The workflow illustrates repeated application of test samples to reconstructed skin tissues, HQ exposure, culture periods, tissue processing, and histological evaluation. Representative tissue sections from nine treatment groups show severe structural disruption and large voids with HQ alone, partial protection with cysteine (Cys) or ascorbic acid (AsA), and greater preservation of tissue architecture in groups containing GDS-23, especially when combined with AsA. The accompanying bar chart quantifies void area ratio (%), with HQ producing the highest damage (~53%) compared with the control (~9%). Treatments containing GDS-23 reduce damage to ~19–20%, while combinations of GDS-23 and AsA, with or without Cys, lower void areas to ~9–11%, near control levels. Statistical annotations indicate significant differences between treatment groups.

**Figure:**
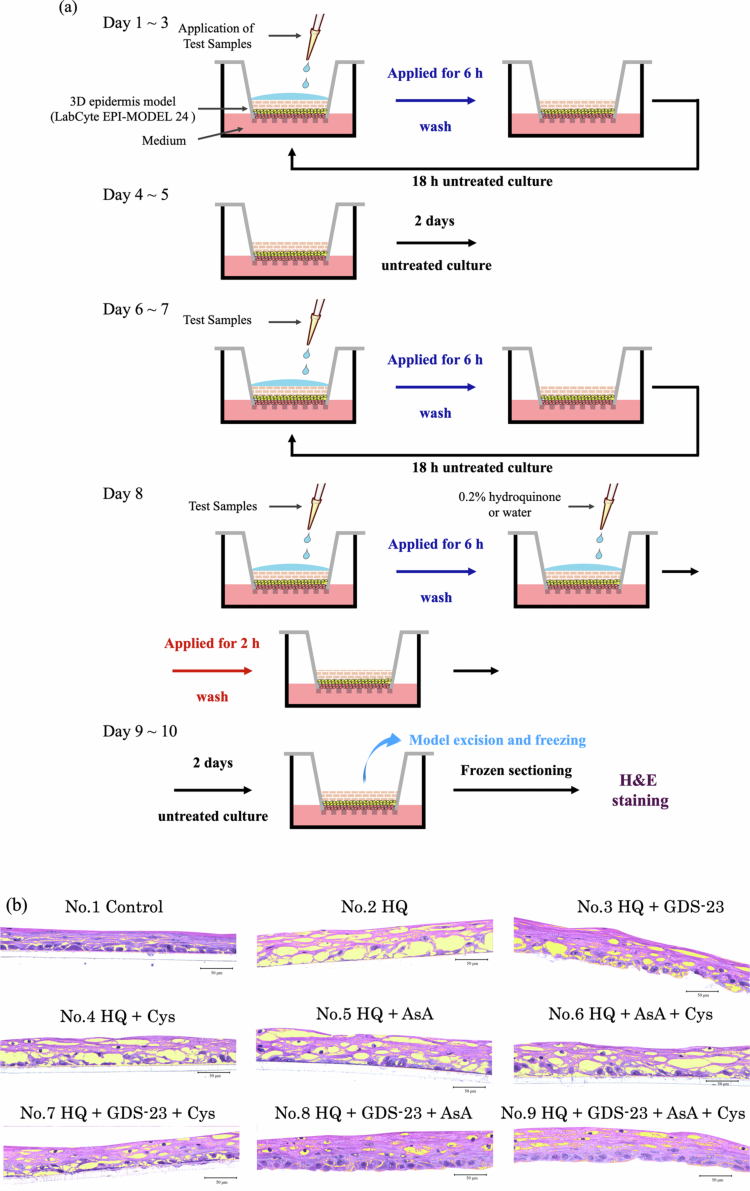

**Figure:**
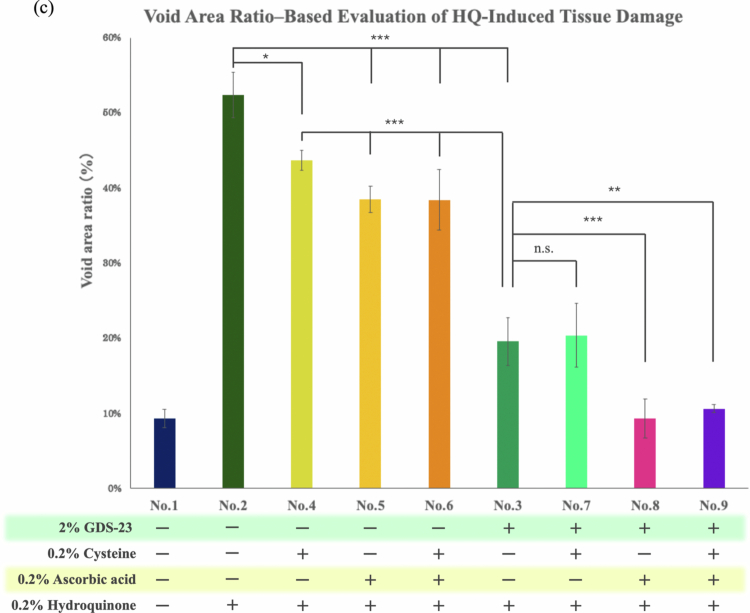

In hydroquinone-only treated tissues, marked structural disruption, including loss of nuclei and collapse of epidermal organisation, accompanied by large void areas characteristic of tissue injury, was observed in the viable epidermal layers (Figure 6b). To quantify damage, the void area ratio within the viable epidermis was calculated (Figure 6c). Tissues treated with empty GDS-23 niosomes exhibited partial preservation of nuclei and reduced void areas compared with that in the hydroquinone-only group, although their morphology remained more disorganised than that of untreated controls. Treatment with cysteine alone, L-ascorbic acid alone, or their combination also attenuated hydroquinone-induced tissue damage, but the void area ratios were higher than those observed in the empty GDS-23 niosome group.

Cysteine-loaded GDS-23 niosomes preserved nuclei and reduced hydroquinone-induced voids to a degree comparable to that in empty GDS-23 niosomes. In contrast, L-ascorbic acid-loaded niosomes produced markedly improved tissue morphology, with nuclear retention and a pronounced reduction in void area approaching that of untreated controls. Niosomes co-loaded with both L-ascorbic acid and cysteine did not show synergistic improvement and exhibited protective effects similar to those observed with L-ascorbic acid-loaded niosomes alone. Collectively, these results indicate that GDS-23 niosomes attenuate hydroquinone-induced tissue injury in the 3D reconstructed epidermis model and that L-ascorbic acid-loaded GDS-23 niosomes exert the most substantial protective effect (Figure 6b,c).

## Discussion

GDS-23, a diacylglycerol–PEG adduct, is a niosome-forming nanocarrier material for DDS. We previously reported that it enhances endogenous antioxidant capacity and upregulates moisture- and barrier-related proteins in the skin via activation of Nrf2 (Miyoshi et al. 2025). In these previous studies, nuclear translocation of Nrf2 was observed, which was accompanied by increased expression of downstream antioxidant proteins, such as NQO1, HO-1, and glutathione-synthesising enzymes, and barrier-related proteins, including filaggrin and loricrin. Furthermore, GDS-23 is an ingredient of cosmetic formulations already in use, and safety evaluations, including a primary skin irritation test conducted in accordance with OECD TG 439 (OECD 2025b), confirmed it categorisation as a non-irritant even at 100% (as-is) (Table S1). In the present study, we focused on the possibility that niosomes formed by GDS-23 possess additional biological activities that can complement and potentiate their carrier function, particularly by modulating intracellular transporters, thereby improving the efficiency of cargo delivery.

The skin forms the outermost surface of the body and is constantly exposed to high oxygen tension, ultraviolet radiation, and various environmental chemicals, making it a key barrier organ that protects the internal milieu from external insults (Proksch et al. 2008; Rinnerthaler et al. 2015). As part of this protective system, antioxidant factors are distributed in a gradient, with higher levels toward the outermost epidermal layers, and the induction of these factors is tightly regulated by the Nrf2–Keap1 signalling pathway (Ishitsuka et al. 2020). This pathway induces classical antioxidant enzymes and contributes to the uptake and regeneration of antioxidant molecules (Harvey et al. 2009; Jaganjac et al. 2020). For example, upregulation of *xCT* promotes the uptake of extracellular cystine, which is reduced intracellularly and used for glutathione synthesis, thereby supporting cellular antioxidant defences (Ye et al. 2014). In contrast, as a consequence of the evolutionary loss of L-gulonolactone oxidase, humans cannot synthesise the antioxidant vitamin L-ascorbic acid (Drouin et al. 2011) and thus rely on exogenous intake followed by transporter-mediated uptake via SVCTs (Savini et al. 2008; May 2011). L-ascorbic acid is widely distributed in various tissues, including the skin, where it mitigates oxidative stress and contributes to cellular protection (Pullar et al. 2017; Michalak et al. 2021). However, the relationship between Nrf2, a master regulator of antioxidant responses, and SVCTs remains unclear. We, therefore, hypothesised that Nrf2 might be involved in regulating SVCT expression and sought to confirm this.

In this study, xCT, a well-established Nrf2-inducible gene and protein (Ye et al. 2014), was used as a reference marker to assess whether SVCT2 exhibits a similar response under Nrf2-activating conditions. We previously confirmed that Nrf2 activation occurs 12–24 h after GDS-23 treatment in NHEKs (Miyoshi et al. 2023). Consistent with this, we observed increased mRNA expression of *xCT* and *SVCT2* beginning at 18 h of GDS-23 treatment. *SVCT1* was also upregulated at the mRNA level (Figure S1), but the level of its expression remained close to the qPCR detection limit and could not be reliably assessed at the protein level; thus, we restricted our evaluation of SVCT1 to gene expression and did not pursue it further. Another diacylglycerol–PEG adduct, GDM-12, also significantly increased the *xCT* and *SVCT2* mRNA levels (Figure S2), indicating that diacylglycerol–PEG adducts, including GDS-23 and GDM-12, generally possess the ability to induce these transporters, with GDS-23 exhibiting the most pronounced effect. In view of these findings, we focused subsequent analyses on GDS-23.

When K67, a compound that suppresses Nrf2 activation by inhibiting the interaction between Keap1 and phosphorylated p62 (Saito et al. 2016), was used, the GDS-23–induced increases in the mRNA expression of *xCT* and *SVCT2* were significantly attenuated. These findings are consistent with previous reports that Nrf2 contributes to *xCT* induction (Ye et al. 2014) and further suggest that Nrf2 may also regulate *SVCT2* expression. However, because only a single Nrf2 inhibitor (K67) was used in the present study, the involvement of the Nrf2 pathway should be interpreted with caution and requires validation in future studies. SVCT2 is a high-affinity L-ascorbic acid transporter that plays a central role in maintaining antioxidant homoeostasis in various tissues, including the skin (Steiling et al. 2007; May 2011; Portugal 2024). Taken together, these data support the hypothesis that Nrf2 contributes to the regulation of *SVCT2* within the antioxidant defence network. Although other pathways might also be involved in the transcriptional control of *SVCT2* (Tian et al. 2016; Portugal 2024), in the present study, we focused on the contribution of the Nrf2 pathway in the context of GDS-23 treatment. Future studies addressing additional transcription factors and signalling pathways will be required to fully elucidate the mechanisms underlying the regulation of L-ascorbic acid transporters. The current findings provide insights into how Nrf2 and *SVCTs* are linked within antioxidant defence systems.

We next examined how GDS-23, which upregulates *xCT* and *SVCT2*, influences drug delivery and intracellular transport efficiency, and whether this contributes to enhanced biological activity. We previously showed that the cytotoxicity of hydroquinone-induced oxidative stress in NHEKs could be attenuated by co-treatment with antioxidants (Miyoshi et al. 2023). We adapted this system to evaluate the combined effects of GDS-23-mediated transporter induction and subsequent exposure to cysteine or L-ascorbic acid. Hydroquinone-induced cytotoxicity occurs through oxidative mechanisms (Nordlund et al. 2006; Draelos 2007; Tentscher et al. 2021). Moreover, our earlier study, as well as the preliminary findings in the present study, confirmed that antioxidants, such as sodium metabisulfite, L-ascorbic acid, and cysteine, reduce hydroquinone cytotoxicity by preventing the oxidation of hydroquinone in the culture medium (Miyoshi et al. 2023) (Figure S3). In the present study, cells in which *xCT* and *SVCT2* had been upregulated by GDS-23 showed significant protection against hydroquinone when low concentrations of cysteine or L-ascorbic acid—ineffective when applied alone—were added. Consistent with our previous findings (Miyoshi et al. 2023), pretreatment with GDS-23 alone enhanced endogenous antioxidant capacity and attenuated hydroquinone-induced cytotoxicity. However, sequential treatment with GDS-23 followed by cysteine or L-ascorbic acid treatment yielded significantly greater cytoprotection than that achieved with GDS-23 alone.

These results suggest that GDS-23-induced upregulation of these transporters may be associated with enhanced intracellular uptake of these substrates, thereby potentially contributing to their protective effects. In particular, SVCT2 exhibits high affinity for L-ascorbic acid, enabling effective transport even at low concentrations (May 2011), whereas SVCT1 shows lower affinity but higher capacity under high L-ascorbic acid conditions (Steiling et al. 2007). The protective effect of L-ascorbic acid is not strictly concentration-dependent, suggesting saturation of SVCT2-mediated uptake at 5–10 µM. The *K*_m_ value of SVCT2 for L-ascorbic acid is approximately 20 µM (Steiling et al. 2007; May 2011; Przybyło and Langner 2020; Portugal 2024). This high-affinity profile is consistent with our hypothesis that uptake may reach a threshold in the lower micromolar range. Overall, these findings suggest that Nrf2-dependent upregulation of *xCT* and *SVCT2* by GDS-23 promotes efficient intracellular uptake of cysteine (cystine) and L-ascorbic acid, thereby enhancing cellular defence against oxidative stress. The combined use of cystine or L-ascorbic acid delivery with transporter induction may thus represent a promising strategy for developing more efficient therapeutic approaches for skin diseases and antioxidant-based interventions.

Based on the findings in the cellular system, we next evaluated GDS-23 niosomes in more physiologically relevant 3D reconstructed human epidermis models. In monolayer cell cultures, where no stratum corneum barrier is present, test substances in the medium act directly on cells, and we did not explicitly confirm niosome formation. However, we previously demonstrated that diacylglycerol–PEG adducts are nonionic surfactants that self-assemble into niosomes (Keller 2006; Keller et al. 2010), and it is reasonable to assume that GDS-23 forms niosomal structures under these conditions. In addition, selection of the test concentration was carefully considered. In monolayer culture systems, because test compounds directly contact NHEKs, relatively low concentrations, such as 50 μM, are sufficient to elicit cellular responses. In contrast, in reconstructed human epidermis models, compounds must penetrate the stratum corneum barrier before reaching viable keratinocytes. Furthermore, the epidermal tissue in the 3D model is maintained by receiver medium located beneath the tissue, and this medium may diffuse into the cellular layers. Consequently, compounds that pass through the stratum corneum diffuse into the medium within the model and become diluted, which reduces the effective concentration to which the cells are exposed. Monolayer culture experiments were, therefore, used as a basic model to evaluate cellular responses that may occur after compounds penetrate the stratum corneum and reach viable epidermal cells. In view of these factors, together with the absence of cytotoxicity in reconstructed human epidermis models reported previously (Miyoshi et al. 2025), safety evaluations including categorisation as a non-irritant in a primary skin irritation test conducted according to OECD TG 439 (OECD 2025b) (Table S1), and considering the concentrations used in topical formulations, GDS-23 was used at a test concentration of 2% in this study. The 3D models used in this study, SkinEthic™ RHE and LabCyte EPI-MODEL 24, contain a stratified structure comprising stratum corneum, granular, spinous, and basal layers and have been reported to reproduce the morphological and physiological characteristics of human epidermis with high fidelity (Hofmann et al. 2023; Katoh et al. 2009b). As these models possess barrier properties similar to those of the native skin, we deemed it essential to verify whether GDS-23 formulations formed niosomes and could penetrate the stratum corneum. Our structural analyses confirmed that GDS-23 could form multilamellar niosomes and that these vesicles enhanced the penetration of encapsulated calcein sodium into the SkinEthic™ RHE model, where fluorescence was detected at significantly higher levels in the epidermis than in aqueous solution controls. Fluorescence microscopy showed signals extending into the deeper stratum corneum and toward the viable epidermis following GDS-23 niosome treatment. However, during nuclear staining with DAPI, the 10-minute incubation and multiple washing steps resulted in partial elution of water-soluble calcein sodium, leading to weaker overall fluorescence. Even under these conditions, fluorescence remained stronger in GDS-23-treated tissues than in controls. To address this, we imaged sections subjected to minimal washing (only for the removal of residual O.C.T. compound) and obtained robust fluorescence from the deep stratum corneum to the vicinity of the viable epidermis in the GDS-23 niosome group compared with that in controls. These observations indicate that GDS-23 niosomes can cross the stratum corneum barrier and reach deeper epidermal layers.

In LabCyte EPI-MODEL 24, immunofluorescence staining showed that xCT and SVCT2 signals increased within DAPI-positive viable epidermal layers following GDS-23 niosome treatment. Taken together with the monolayer and SkinEthic™ RHE data, these results suggest that GDS-23–derived niosomes penetrate the stratum corneum, reach the viable epidermis, and exert pharmacological effects leading to transporter induction. The observed increase in *xCT* and *SVCT2* expression in the viable layers of GDS-23–treated 3D models supports the notion that GDS-23 retains its niosomal structure during penetration and acts at the epidermal cell level. Niosomes with multilamellar structures traverse the intercellular lipid pathway or partially fuse with stratum corneum lipids, allowing for deeper delivery of the encapsulated cargo without severely disrupting the barrier integrity (Niu et al. 2019; Zoabi et al. 2021; Akombaetwa et al. 2023). Moreover, we previously reported that GDS-23 activates the Nrf2 pathway and increases the expression of barrier-related proteins, such as filaggrin, loricrin, and aquaporin-3 (Miyoshi et al. 2025). Increased signals of these marker proteins were also confirmed via immunofluorescence staining in reconstructed human epidermis, indicating that GDS-23 does not impair the epidermal barrier integrity and may instead support the barrier function. Our findings are in line with this concept and suggest that GDS-23 niosomes partially lose outer lamellae during passage but still reach viable epidermal cells in a structurally intact and functionally active form.

We speculate that GDS-23, once at the viable layer, may interact with membrane lipids and initiate intracellular signalling. Structurally, GDS-23 contains a diacylglycerol (DAG) moiety, which activates protein kinase C (PKC) isoforms, leading to phosphorylation of downstream substrates and modulation of diverse signalling cascades (Bell 1986; Kazanietz and Cooke 2024). It is, therefore, plausible that the DAG structure within GDS-23 interacts with keratinocyte membrane lipids, may activate PKCδ, and subsequently promote p62 phosphorylation and Nrf2 activation (Park et al. 2021), ultimately inducing *xCT* and *SVCT2* expression. However, the precise route by which GDS-23 niosomes reach and act on viable epidermal cells remains to be elucidated, and further mechanistic studies will be required.

We also investigated the delivery of cysteine and L-ascorbic acid encapsulated in GDS-23 niosomes, as well as the contribution of *xCT* and *SVCT2* upregulation to intracellular transport efficiency, using LabCyte EPI-MODEL 24. Hydroquinone-induced oxidative tissue damage was again used as a functional readout, and H&E staining of sections including the basal layer was used to assess the extent of injury. H&E staining, which differentially colours nuclei (blue-purple) and cytoplasm/extracellular matrix (pink to red), is widely used to visualise structural changes in the stratum corneum, granular, spinous, and basal layers, as well as necrosis, cell loss, void formation, inflammation, and fibrosis in skin and other tissues (Haggerty et al. 2014).

As the principal effects of GDS-23 involve Nrf2 activation, endogenous antioxidant enhancement, and *xCT/SVCT2* induction within viable layers, we focused our quantitative analysis on damage to the viable epidermis. Hydroquinone-treated tissues exhibited loss of nuclei, pronounced cell loss, and structural collapse within viable layers, especially around the basal layer, accompanied by numerous void regions that were interpreted as areas of tissue damage (Owolabi et al. 2020). To quantify injury, we calculated the proportion of void area within the viable epidermis as a ‘void ratio.’ Treatment with GDS-23 niosome alone reduced hydroquinone-induced damage, preserved nuclei, and significantly lowered the void ratio compared with that in tissues exposed only to hydroquinone, consistent with the previously reported antioxidant effects of GDS-23 (Miyoshi et al. 2023). Nevertheless, tissue architecture in the GDS-23 group remained somewhat disorganised compared with that in untreated controls, and some voids persisted, suggesting incomplete protection against oxidative stress. Treatment with cysteine alone, L-ascorbic acid alone, or their combination also reduced hydroquinone-induced void formation, likely reflecting their inherent antioxidant activities (Pullar et al. 2017; Ezeriņa et al. 2018; Kawashima et al. 2018). When cysteine- or L-ascorbic-acid–loaded GDS-23 niosomes were applied, preservation of nuclei and tissue structure in the viable epidermis was more evident. These comparisons allow partial discrimination between the intrinsic antioxidant effects of the compounds and the additional contribution of GDS-23 niosomal delivery. Among the tested conditions, L-ascorbic acid-loaded niosomes provided the most pronounced protection, as evidence in H&E images, and markedly decreased the void ratio, whereas cysteine-loaded niosomes produced relatively favourable morphology but a void ratio comparable to that observed with GDS-23 niosomes alone. The differential behaviour of cysteine and L-ascorbic acid may reflect differences in their uptake routes and antioxidant modes of action: cysteine is predominantly present as extracellular cystine; it is taken up via xCT and is reduced intracellularly before contributing to antioxidant defences through multiple pathways including glutathione synthesis (Habib et al. 2015), whereas L-ascorbic acid is directly transported into cells via SVCT2 and exerts antioxidant effects within the cytosol (Pullar et al. 2017). As an additional observation, in the L-ascorbic-acid-loaded niosome-treated models, a subset of cells exhibited morphological changes resembling ‘sunburn cells,’ such as nuclear condensation and cytoplasmic shrinkage, which are typically observed under UVB-induced oxidative stress (Van Laethem et al. 2005; Gao et al. 2021). This suggests that hydroquinone-induced oxidative damage was not completely suppressed. Nevertheless, the void ratio in this group was comparable to that in the untreated control. Thus, although the void ratio has limitations as a sole metric for precisely evaluating the extent of tissue damage, it remains a useful indicator for quantitatively comparing structural alterations within the viable epidermal layers. In this study, the void ratio was used as the primary quantitative parameter to assess whether enhanced tissue-protective effects were associated with DDS-mediated delivery.

The present results indicate that relying exclusively on void-based quantification may not fully reflect the degree of oxidative injury. Therefore, future studies should establish a multifaceted evaluation system that integrates histomorphological assessment with additional assays, such as the Neutral Red uptake assay. In this study, we primarily focused on tissue damage in the viable epidermal layers; however, hydroquinone treatment also induced detachment within the stratum corneum (Figure S5). This detachment might have resulted from oxidation of residual hydroquinone in the stratum corneum, followed by structural weakening and denaturation of corneodesmosomes and corneocyte-adhesion proteins by its oxidative metabolites and reactive oxygen species, ultimately promoting protease-mediated degradation (Thiele et al. 1999; Borgia et al. 2022). As investigation of this phenomenon was not the primary focus of the present study, it was not pursued further. This study also had certain mechanistic limitations. In this study, increased protein signals of xCT and SVCT2 were observed via immunofluorescence in the 3D reconstructed epidermis model following gene expression analysis in NHEKs; however, quantitative protein analysis such as western blotting was not performed. The role of Nrf2 in *SVCT2* upregulation was suggested based only on inhibitor experiments and no direct evidence was obtained, such as through promoter-binding analysis.

In addition, because only a single Nrf2 inhibitor (K67) was used in the present study, the involvement of the Nrf2 pathway should be interpreted with caution. Moreover, the potential effects of sustained or long-term Nrf2 activation were not examined and remain unclear.

Additionally, all experiments were conducted with NHEKs or reconstructed epidermal models without verification in other skin cell types. Furthermore, reconstructed epidermis models do not fully reproduce the biological complexity of the human skin and lack several physiological components present in vivo, including skin appendages and cellular heterogeneity (Neupane et al. 2020). Future research should aim to address these limitations.

Taken together, our results suggest that niosomes formed by GDS-23 can penetrate the stratum corneum, reach the viable epidermis, and upregulate *xCT* and *SVCT2* expression, thereby potentially contributing to increased intracellular uptake and accumulation of cystine and L-ascorbic acid. These events are associated with the attenuation of oxidative stress–induced tissue damage in both monolayer and 3D skin models. The data indicate that GDS-23-derived niosomes not only function as physical carriers that cross the skin barrier but also act as bioactive materials that modulate transporter expression and enhance the intracellular delivery of encapsulated antioxidants. Furthermore, our findings support a potential role for Nrf2 as a regulator of SVCT2, a transporter central to antioxidant homoeostasis, thereby providing new insights into the control of L-ascorbic acid uptake. Overall, GDS-23 may serve as a DDS material with the potential to synergistically enhance antioxidant delivery, transporter induction, and intracellular antioxidant accumulation. As such, GDS-23 is expected to contribute to the development of new therapeutic strategies for skin diseases and oxidative stress–related conditions.

## Conclusions

This study demonstrates that niosomes formed by the diacylglycerol–PEG adduct GDS-23 possess dual functionality as a DDS. In particular, it (i) facilitates the delivery of encapsulated molecules across the stratum corneum barrier through enhanced transdermal penetration and (ii) may promote intracellular uptake in viable epidermal layers by upregulating transporter expression. Based on the combined contribution of these two mechanisms, GDS-23 niosomes are expected to function as an effective and versatile DDS platform for cutaneous delivery.

To the best of our knowledge, this study is the first to suggest that Nrf2 activation contributes to *SVCT2* upregulation. Moreover, the combined effects of drug delivery via GDS-23 niosomes and transporter induction were reproduced in both cell-based assays and three-dimensional skin models. Among these effects, the combination of L-ascorbic acid delivery and *SVCT2* induction produced particularly strong tissue-protective activity, supporting the notion that SVCT2 is a highly efficient transporter. These findings suggest that GDS-23 niosomes enhance the efficiency of utilisation of L-ascorbic acid delivered into the skin.

Taken together, GDS-23 is a promising multifunctional DDS nanocarrier that simultaneously enables transdermal delivery of antioxidant molecules via niosomes and may enhance intracellular uptake through Nrf2-mediated induction of *xCT* and *SVCT2*. This dual functionality of GDS-23 opens new avenues for therapeutic applications in skin disorders and antioxidant-based treatments.

## Supplementary Material

TableS1.tif

FigureS5.tif

FigureS4b.tif

FigureS4a.tif

FigureS3.tif

FigureS2.tif

FigureS1.tif

Supplementary figure table caption Supplementary figure table caption

## Data Availability

The data that support the findings of this study are available from the corresponding author upon reasonable request.
