# Supplementary figures and images for: Dual-mechanism vitamin C delivery by polyethylene glycol-23 glyceryl distearate-based niosomes via SVCT2 induction and enhanced transdermal penetration
Source: Drug Deliv. 2026 May 30;33(1):2681287. doi: 10.1080/10717544.2026.2681287 (PMC13224707; doi:10.1080/10717544.2026.2681287)

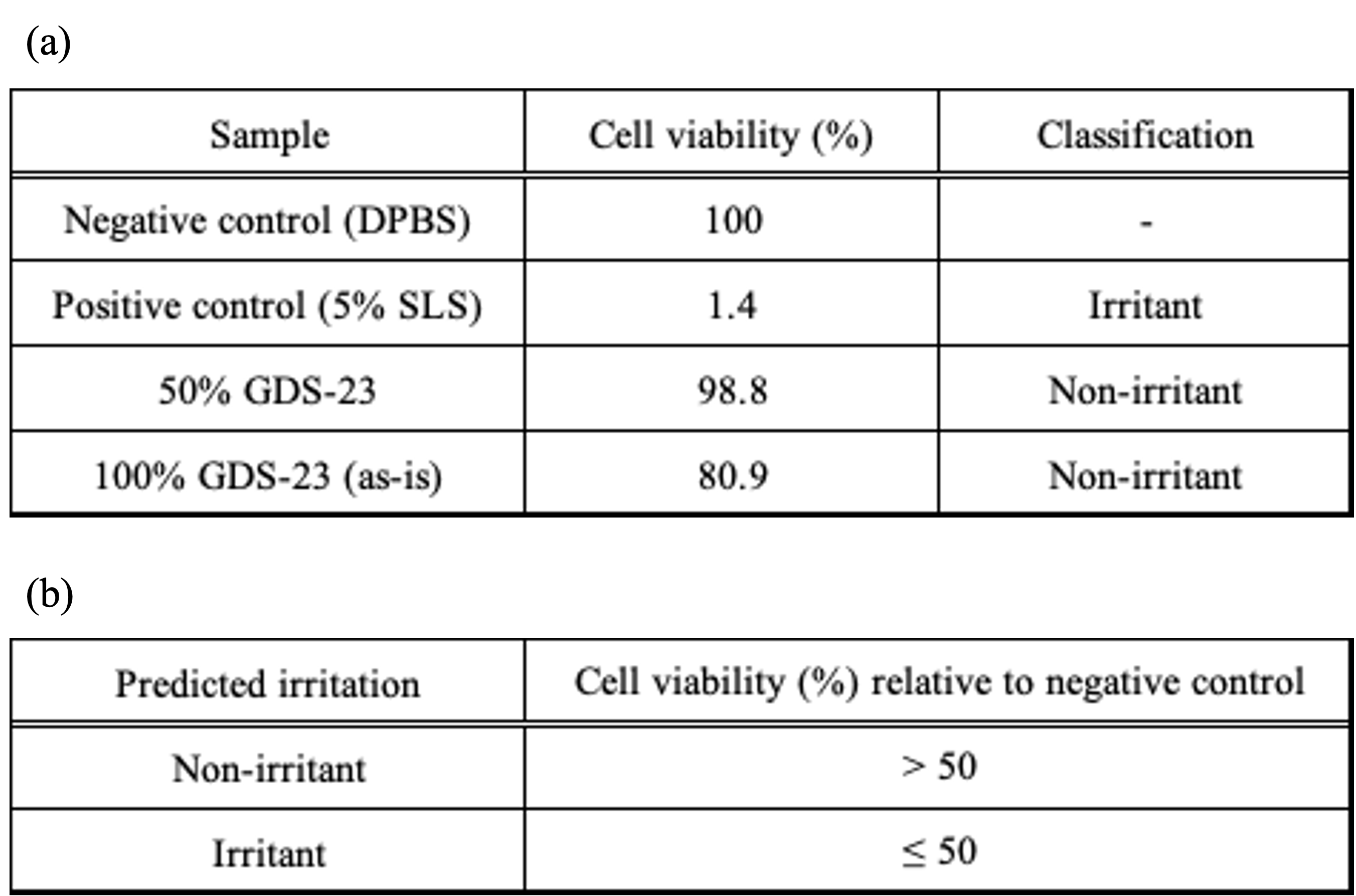

Supplement: TableS1.tif [file IDRD_A_2681287_SM3367.tif]

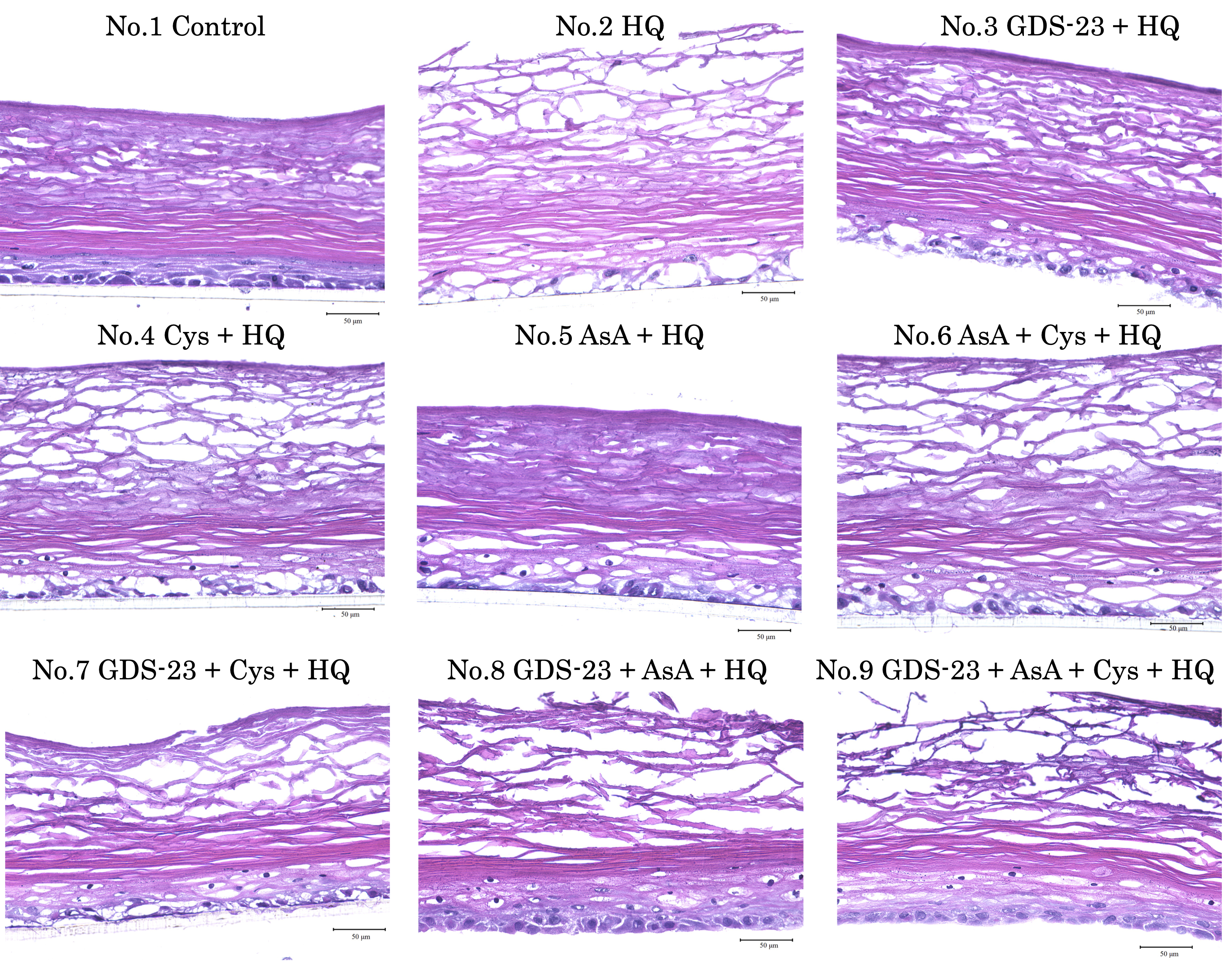

Supplement: FigureS5.tif [file IDRD_A_2681287_SM3361.tif]

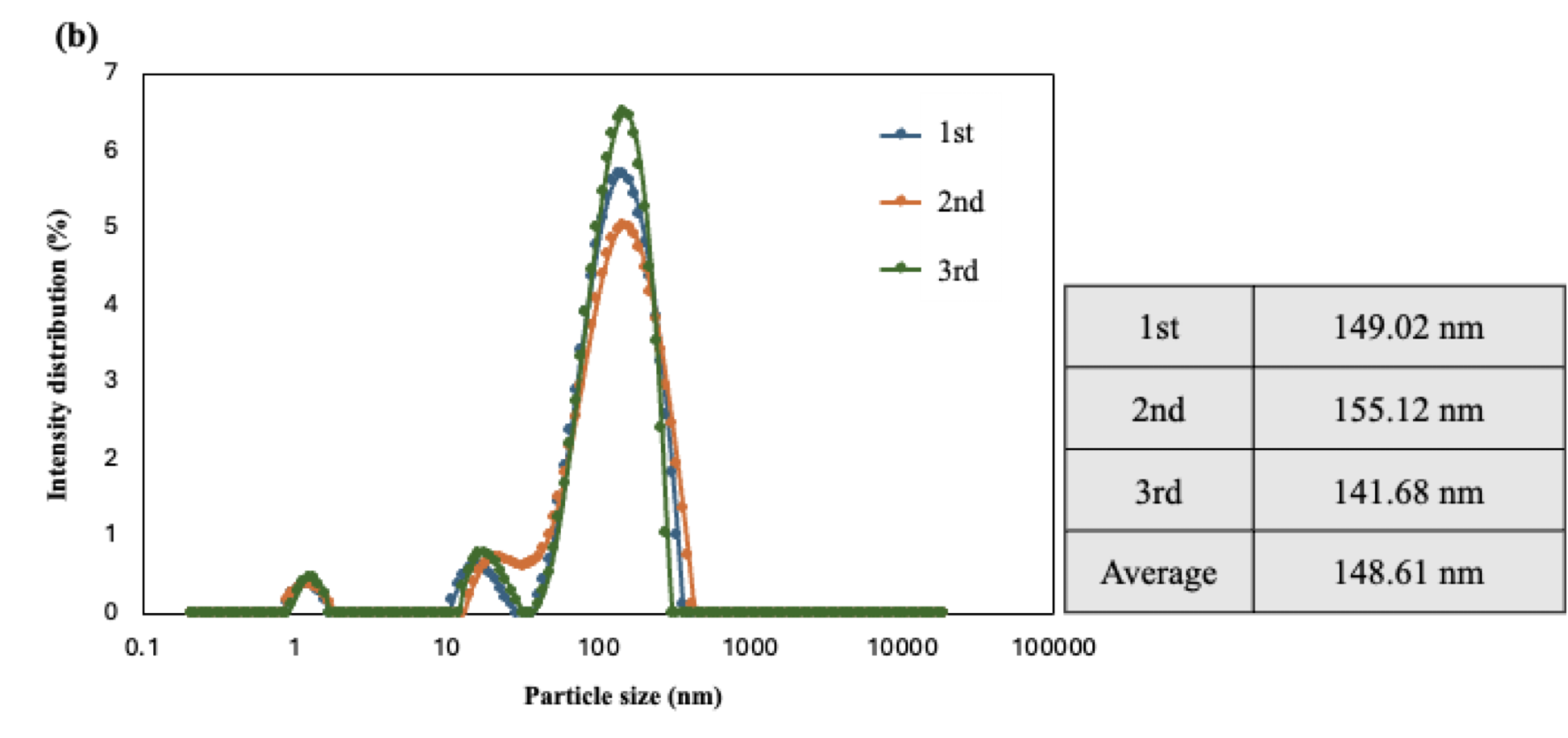

Supplement: FigureS4b.tif [file IDRD_A_2681287_SM3360.tif]

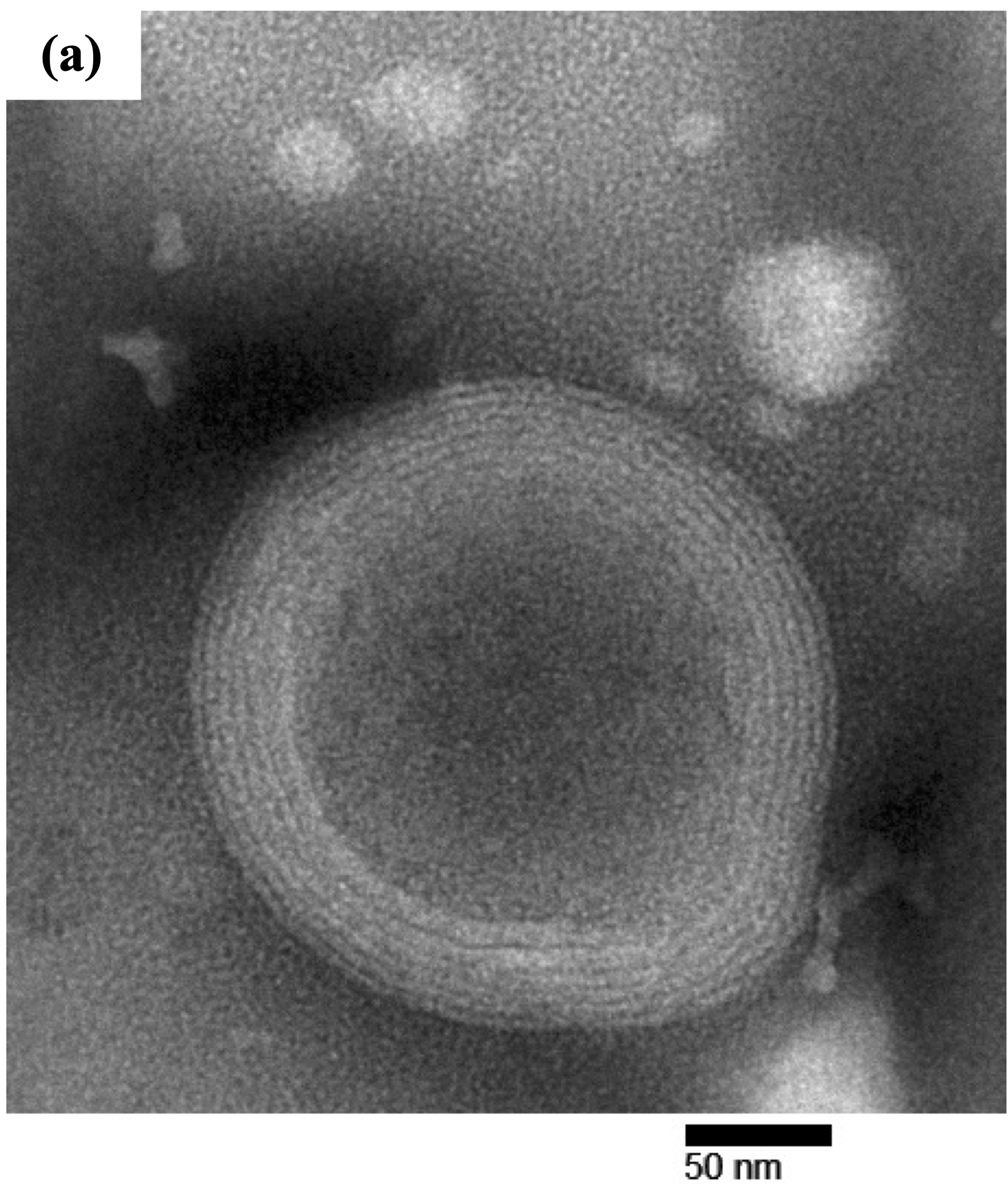

Supplement: FigureS4a.tif [file IDRD_A_2681287_SM3359.tif]

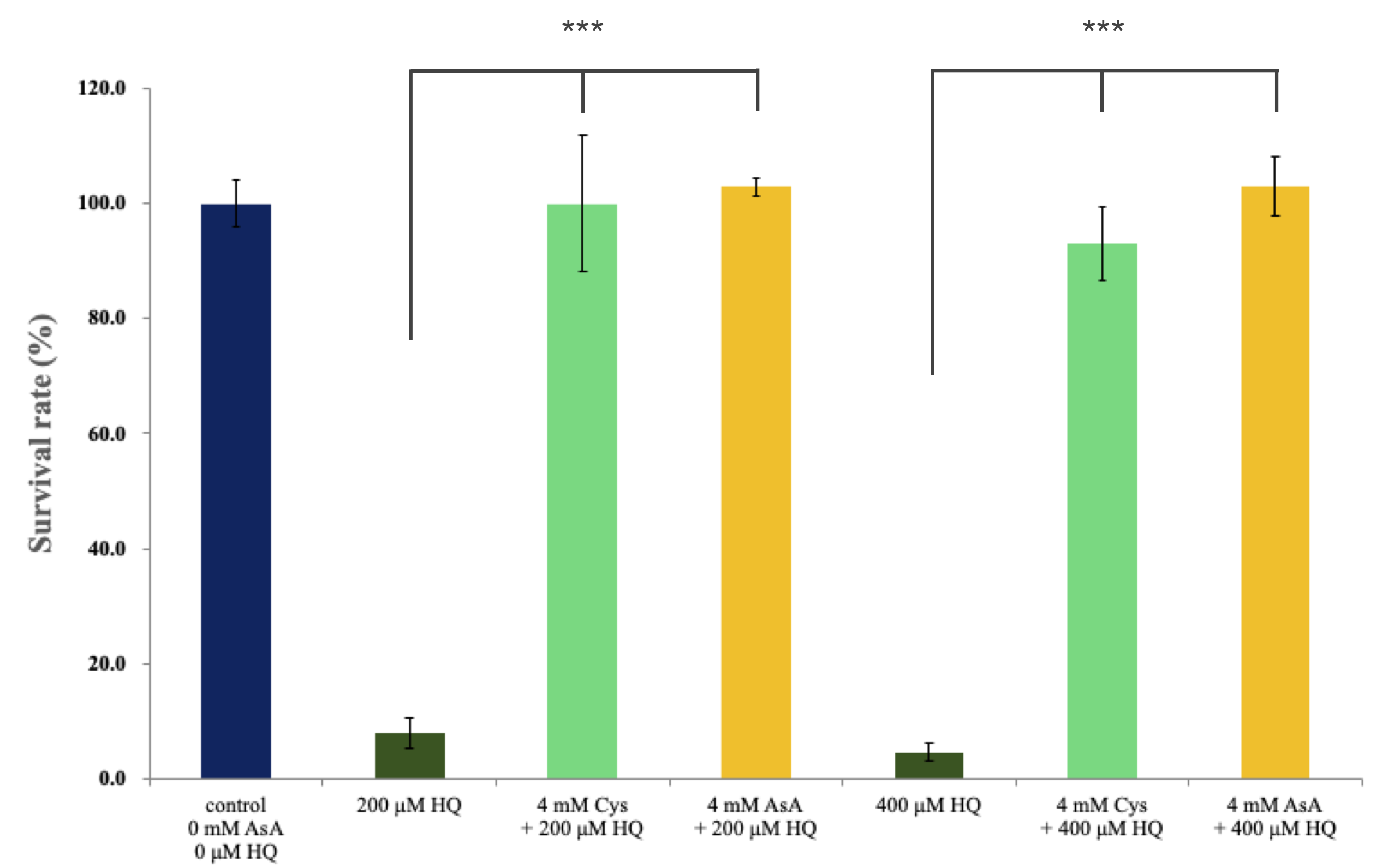

Supplement: FigureS3.tif [file IDRD_A_2681287_SM3339.tif]

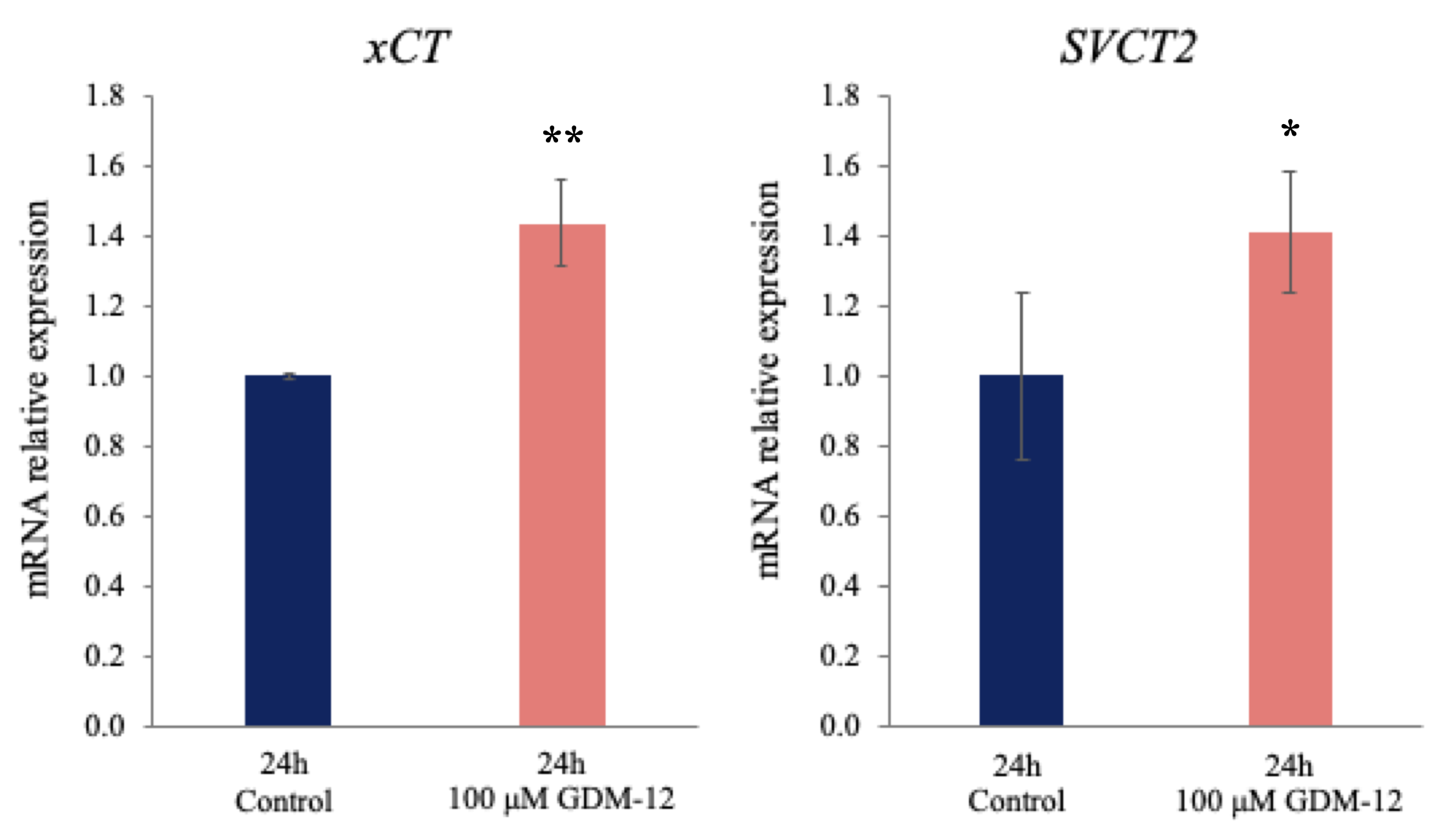

Supplement: FigureS2.tif [file IDRD_A_2681287_SM3294.tif]

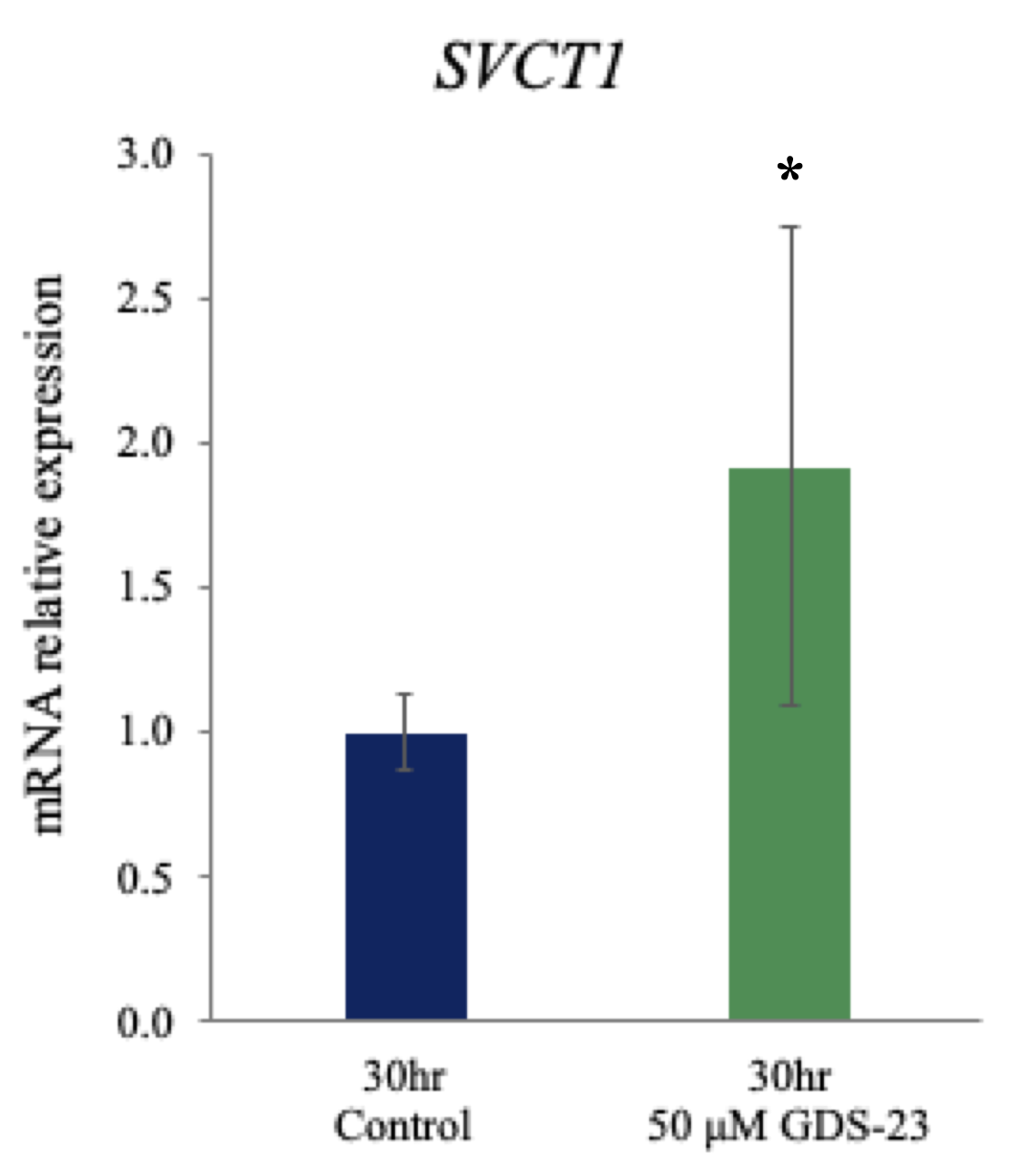

Supplement: FigureS1.tif [file IDRD_A_2681287_SM3277.tif]
