# Supplementary material for: Dual-mechanism vitamin C delivery by polyethylene glycol-23 glyceryl distearate-based niosomes via SVCT2 induction and enhanced transdermal penetration
Source: Drug Deliv. 2026 May 30;33(1):2681287. doi: 10.1080/10717544.2026.2681287 (PMC13224707; doi:10.1080/10717544.2026.2681287)
Supplement: Supplementary figure table caption [file IDRD_A_2681287_SM5893.docx]

**Supplementary Materials**

**Figure S1. Effect of GDS-23 on *SVCT1* mRNA expression.**

NHEKs were treated with 50 µM GDS-23 for 24 h, followed by incubation in culture medium alone for an additional 6 h (total 30 h). Control cells were incubated with culture medium alone. *SVCT1* mRNA levels were quantified using quantitative PCR (qPCR). Data are presented as mean ± standard deviation (n = 4). Statistical analysis was performed using Student’s *t*-test (*p < 0.05, **p < 0.01, and ***p < 0.001 vs. control).

**Figure S2. Effect of polyethylene glycol-12 glyceryl dimyristate (GDM-12) on *xCT* and *SVCT2* mRNA expression.**

NHEKs were treated with 100 µM GDM-12 for 24 h. Control cells were incubated with culture medium alone. mRNA levels of *xCT* and *SVCT2* were quantified using quantitative PCR (qPCR). Data are presented as mean ± standard deviation (n = 4). Statistical analysis was performed using Student’s *t*-test (*p < 0.05, **p < 0.01, and ***p < 0.001 vs. control).

**Figure S3. Preliminary assessment of hydroquinone-induced cytotoxicity in normal human dermal fibroblasts (NHDFs).**

NHDFs were treated with 200 or 400 µM hydroquinone alone or co-treated with 4 mM cysteine or 4 mM L-ascorbic acid, and the extent of cytotoxicity was compared among groups. Cytotoxicity was evaluated using the neutral red assay and expressed as the percentage of viability relative to control cells. Data are presented as mean ± standard deviation (n = 6). Statistical analysis was performed using Dunnett’s test (*p < 0.05, **p < 0.01, and ***p < 0.001).

**Figure S4. Structural characterization of 2% GDM-12 niosomes and percutaneous absorption assessment using a 3D-cultured epidermal model (SkinEthic™ RHE).**

(a) Transmission electron microscopy (TEM) images. (b) Particle size distribution measured via dynamic light scattering (DLS). (c) Percutaneous absorption assay (quantitative analysis): From the stratum corneum side of the SkinEthic™ RHE tissues, 150 µL of either 2.0% GDM-12 aqueous solution containing 0.1% calcein sodium or 0.1% calcein sodium aqueous solution was applied and incubated for 6 h. After treatment, calcein sodium was extracted from the tissues, and fluorescence intensity (excitation: 494 nm, emission: 520 nm) was measured. The amount of permeated calcein sodium was determined from a standard calibration curve, and the results were expressed as a ratio to the control. Data are presented as mean ± standard deviation (n = 4). Statistical significance was determined using Student’s *t-*test (*p < 0.05, **p < 0.01, ***p < 0.001 vs. control).

**Figure S5. Overview of hematoxylin and eosin (H&E)-stained sections from 3D-cultured epidermal tissues (LabCyte EPI-MODEL 24) treated with 2% GDS-23 in the evaluation of hydroquinone-induced tissue damage.**

Shown are whole-section H&E images of three-dimensional cultured epidermal tissues (LabCyte EPI-MODEL 24) processed according to the experimental design described in the protocol.

**Table S1. Primary skin irritation test of GDS-23 according to OECD TG 439.**

(a) Methods and results: Skin irritation was evaluated according to OECD TG 439 using a reconstructed human epidermis model (LabCyte EPI-MODEL24). Test substances (25 μL) were applied to the tissue surface for 15 min, followed by washing with DPBS (−) and incubation for 42 h. Cell viability was determined using the MTT assay, and absorbance at 570 nm was corrected for background absorbance at 650 nm. The cell viability values for all test conditions were greater than 50% relative to the negative control, and therefore the test material was classified as non-irritant according to the acceptance criteria. (b) Classification criteria.

Abbreviations: DPBS (−), Dulbecco’s phosphate-buffered saline without Ca²^+^ and Mg²^+^; SLS, sodium lauryl sulfate; MTT, 3-(4,5-dimethylthiazol-2-yl)-2,5-diphenyltetrazolium bromide.
